# Downregulation of *PARP1* transcription by promoter-associated E2F4-RBL2-HDAC1-BRM complex contributes to repression of pluripotency stem cell factors in human monocytes

**DOI:** 10.1038/s41598-017-10307-z

**Published:** 2017-08-25

**Authors:** Ewelina Wiśnik, Tomasz Płoszaj, Agnieszka Robaszkiewicz

**Affiliations:** 1 Department of Biophysics of Environmental Pollution, Institute of Biophysics, University of Lodz, Pomorska 141/143, 90-236 Lodz, Poland; 2 Department of Molecular Biology, Medical University of Lodz, Narutowicza 60, 90-136 Lodz, Poland; 3 Department of General Biophysics, Institute of Biophysics, University of Lodz, Pomorska 141/143, 90-236 Lodz, Poland

## Abstract

Differentiation of certain cell types is followed by a downregulation of PARP1 expression. We show that the reduction in the abundance of PARP1 in hematopoietic progenitor cells and monocytes is tightly controlled by the cell cycle. The differentiation-associated cell cycle exit induces E2F1 replacement with E2F4 at the *PARP1* promoter and the assembly of an E2F4-RBL2-HDAC1-BRM(SWI/SNF) repressor complex which deacetylates nucleosomes and compacts chromatin. In G1 arrested cells, *PARP1* transcription is reduced by the recruitment of E2F1-RB1-HDAC1-EZH2(PRC2)-BRM/BRG1(SWI/SNF), which additionally trimethylates H3K27 and causes an even higher increase in nucleosome density. The re-establishment of an active chromatin structure by treating post-mitotic monocytes with the HDAC inhibitor and G1 arrested cells with a combination of HDAC and EZH2 inhibitors restores *PARP1* expression completely but does not affect the interaction between the components of the repressor complex with chromatin. This suggests that RB1 and RBL2, as well as PRC2, SWI/SNF and HDAC1, do not interfere with the transcription machinery. Interestingly, reinstatement of PARP1 expression by the silencing of RBL2 or by the inhibition of HDACs in monocytes and by transfection with the PARP1 expression vector in differentiated THP-1 cells substantially increased transcription of pluripotency stem cell factors such as POU5F1, SOX2 and NANOG.

## Introduction

Although PARP1 is involved in the regulation of numerous intracellular processes such as DNA repair, gene transcription, signalling or metabolism, the differentiation of certain cell types is associated with downregulation of *PARP1* transcription^1,2^. Decreased abundance of PARP1 also occurs in human monocytes derived from hematopoietic progenitor and stem cells (HSPCs), which belong to a group of multipotent cells capable of self-renewal and, upon stimulation, of giving rise to a wide range of blood cells. Lineage commitment in HPSC caused by cytokines or cell-cell signalling, involves the inhibition of cell cycle progression, repression of HPSC specific transcription factors and induction of lineage-specific expression of genes involved in cell fate. For example, PU.1 (also known as SPI-1) acts in monocytes/macrophages as a lineage-determining transcription factor^3^. Neither the mechanism nor the physiological significance of *PARP1* repression in determining monocyte phenotype, function or differentiation has been documented. The low level of this enzyme has been shown to sensitise human monocytes to oxidative stress, while in myotubes it served as a protective mechanism against oxidative stress, helping with maintaining the cellular functions of skeletal muscles^4,5^. According to recent findings *PARP1* repression favours commitment and differentiation of some cell types. In differentiating osteoclasts, PARP1 was demonstrated to act as a repressor of osteoclastogenesis-promoting factors such as *NFATc1* and *IL1β*^6^. Furthermore, PARP1 might be considered as another DNA-interacting transcription cofactor sustaining cell stemness. Roper *et al*. showed that PARP1 safeguards embryonic stem cell pluripotency by occupying the promoters of *NANOG*, *POU5F1*, *SELLA* and *ZFP42* and by maintaining an active chromatin configuration (reduced H3K9me3 and H3K27me3 as well as DNA methylation), thereby sustaining the transcription of above mentioned genes^9^. Similarly, ADP-ribosylation of SOX2 by PARP1 was required for the dissociation of inhibitory SOX2 from the enhancer of proliferation-promoting fibroblast growth factor FGF4 in embryonic stem cells^7^.

Findings coming from a differentiation model, in which PARP1 deficiency induced ES cells to differentiate into trophectodermal cells as well as into derivatives of all three germ layers in embryoid bodies, are in line with the concept of PARP1’s role in the maintenance of pluripotency^8,9^.

Current knowledge on the regulation of *PARP1* transcription is limited to very few papers which describe selected cases but, at the same time, underline the complex nature of the possible modulation of *PARP1* expression, including DNA modification, presence of transcription factors associated with chromatin as well as cell type-specific miRNA availability. Since the human *PARP1* promoter overlaps the CpG island, recent toxicological papers have linked *PARP1* repression to methylation of its promoter and activation of DNA methyltransferase 1 (DNMT1) in cells exposed to nano-silicon dioxide (nano-SiO2) and benzene^10,11^. Another possible mechanism of *PARP1* regulation was revealed in the culture of rat and rabbit primary cells, where *PARP1* transcription was influenced by cell density and the SP1 transcription factor, which suggested the possible association of *PARP1* expression with cell proliferation and cell cycle progression^12^. Chromatin-independent mechanisms of PARP1 mRNA abundance regulation were attributed to the action of miR-223 which targeted the PARP1 transcript in oesophageal adenocarcinoma cells^13^.

In this study, we show that PARP1 is less abundant in differentiated monocytes than in cultured, proliferating CD34+ hematopoietic progenitor and stem cells and that downregulation of *PARP1* transcription facilitates repression of pluripotent transcription factors in human monocytes. Moreover, we provide a description of the complete mechanism which links *PARP1* transcription with monocyte differentiation and the cell cycle exit. In this model, RISC- and DNA methylation-independent downregulation of *PARP1* transcription involves the retinoblastoma family of DNA-interacting proteins, which together with corresponding E2F transcription factors assemble other nucleosome remodelling enzymes (HDAC1, EZH2 – PRC2, BRM/BRG1 – SWI/SNF) at the *PARP1* promoter in a cell cycle-dependent manner.

## Results and Discussion

### PARP1 repression in human monocytes is associated with a decrease in the transcription of pluripotency transcription factors and RUNX1, GATA2 and PAX5

**Figure 1:**
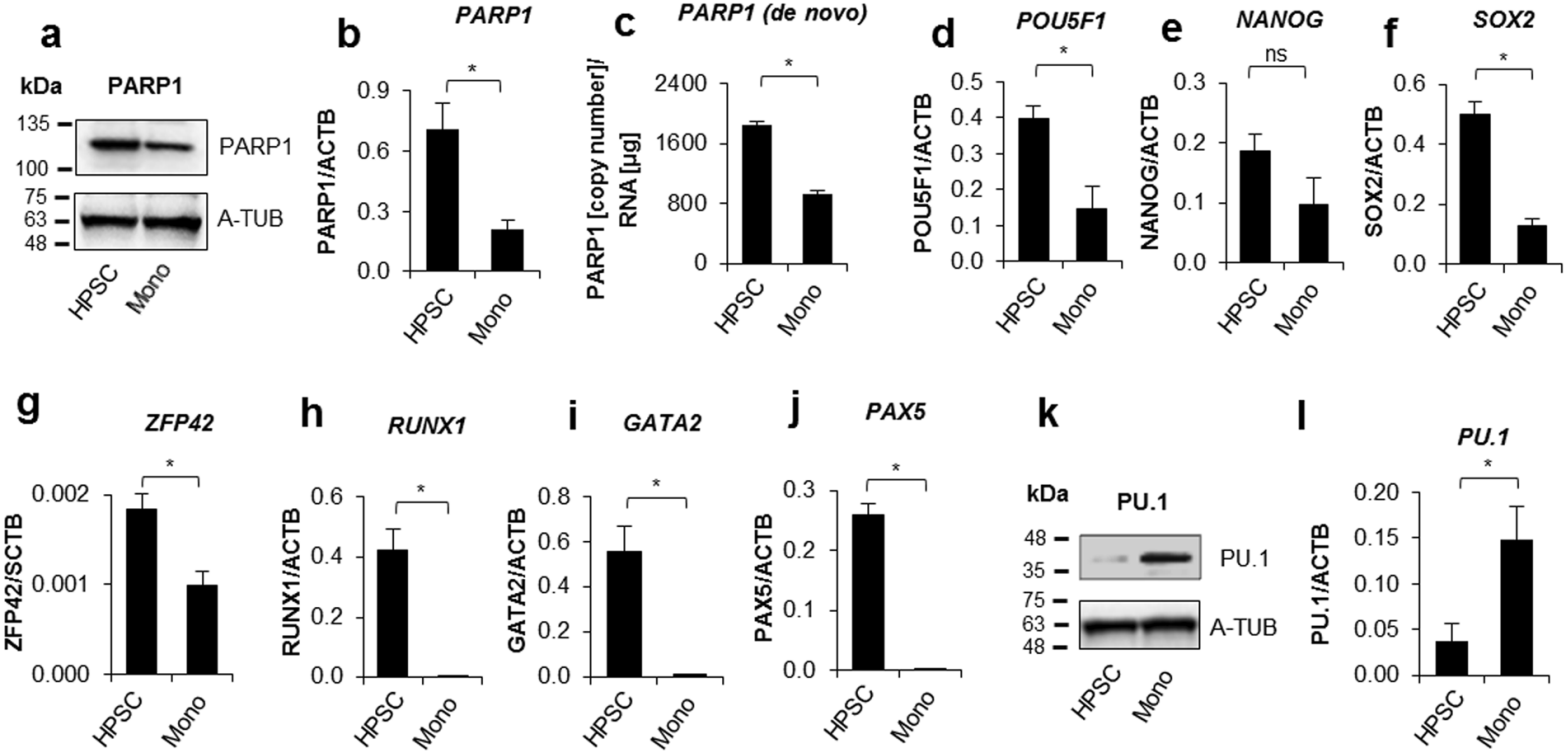
Downregulation of stem cell transcription factors is associated with *PARP1* repression. The expression of PARP1, selected transcription factors as well as PU.1/SPI-1 was compared between human hematopoietic stem and progenitor cells (HSPC) and monocytes with *real-time* PCR (**b**,**d–j**,**l**) and, additionally, PARP1 and PU.1 protein was determined using western blot (**a** and **k**). The accumulation of nascent mRNA (**c**) in HPSC and monocytes was monitored using Click-iT® Nascent RNA Capture Kit (Thermofisher Scientific).

Bearing in mind that previous reports documented the low abundance of PARP1 in human monocytes we first examined if PARP1 repression occurs in differentiated CD14+ blood-derived cells (monocytes represented over 90% of cells, Supplem. Figure 1a) or it is also observed in proliferating CD34+ umbilical cord blood-derived hematopoietic progenitor and stem cells (HPSC, Thermofisher Scientific). As is shown in Fig. 1a and b both the level of PARP1 protein and mRNA were substantially lower in monocytes. The accumulation of nascent RNA over a period of 12 h of cell culture was also diminished in differentiated CD14+ cells (Fig. 1c), suggesting that PARP1 availability in monocytes is reduced by limited mRNA synthesis or augmented degradation. To track monocyte differentiation-associated changes in two closely related cell status, we introduced another cell line: proliferating THP-1 premonocytes committed to monocytic cell lineage by the treatment with PMA. The same pattern of changes in PARP1 expression was observed during PMA-induced differentiation of THP-1 cells (Supplem. Figure 1b–d)^14^.

Since recent data have indicated that PARP1 interferes, in particular cases, with cell differentiation by sustaining transcription of pluripotency genes, we examined if some of these genes are differentially expressed in cultured HPSCs, which are documented to express known embryonic stem cell factors^15^, and in monocytes, which show a lower abundance of PARP1. As is shown in Fig. 1d–g, only the mRNA level of *NANOG* remained unchanged, while the expression of all other markers of pluripotency (*POU5F1*, *SOX2*, *ZFP42*) was substantially downregulated in monocytes. Similarly, other three transcription factors highly transcribed in HPSCs: *RUNX1* and *GATA2* (maintaining HPSC status) and *PAX5* (associated with B cell differentiation) were strongly repressed in monocytes (Fig. 1h–j). To validate the specificity of *real-time* PCR used for mRNA quantification, reverse transcribed cDNA templates were additionally sequenced (Supplem. Figure 1e–k).

In contrast to HPSCs, monocytes showed strong expression of the lineage-determining factor PU.1 (Fig. 1k and l; similarly in differentiated THP-1, Supplem. Figure 3f and g) which, confirms the approved concept of the interdependence and negative correlation between expression of pluripotency and cell type-specific genes during cell differentiation^16,17^.

### Repression of PARP1 transcription is independent of CpG island methylation, SP1 and PU.1 interaction with the PARP1 promoter

**Figure 2:**
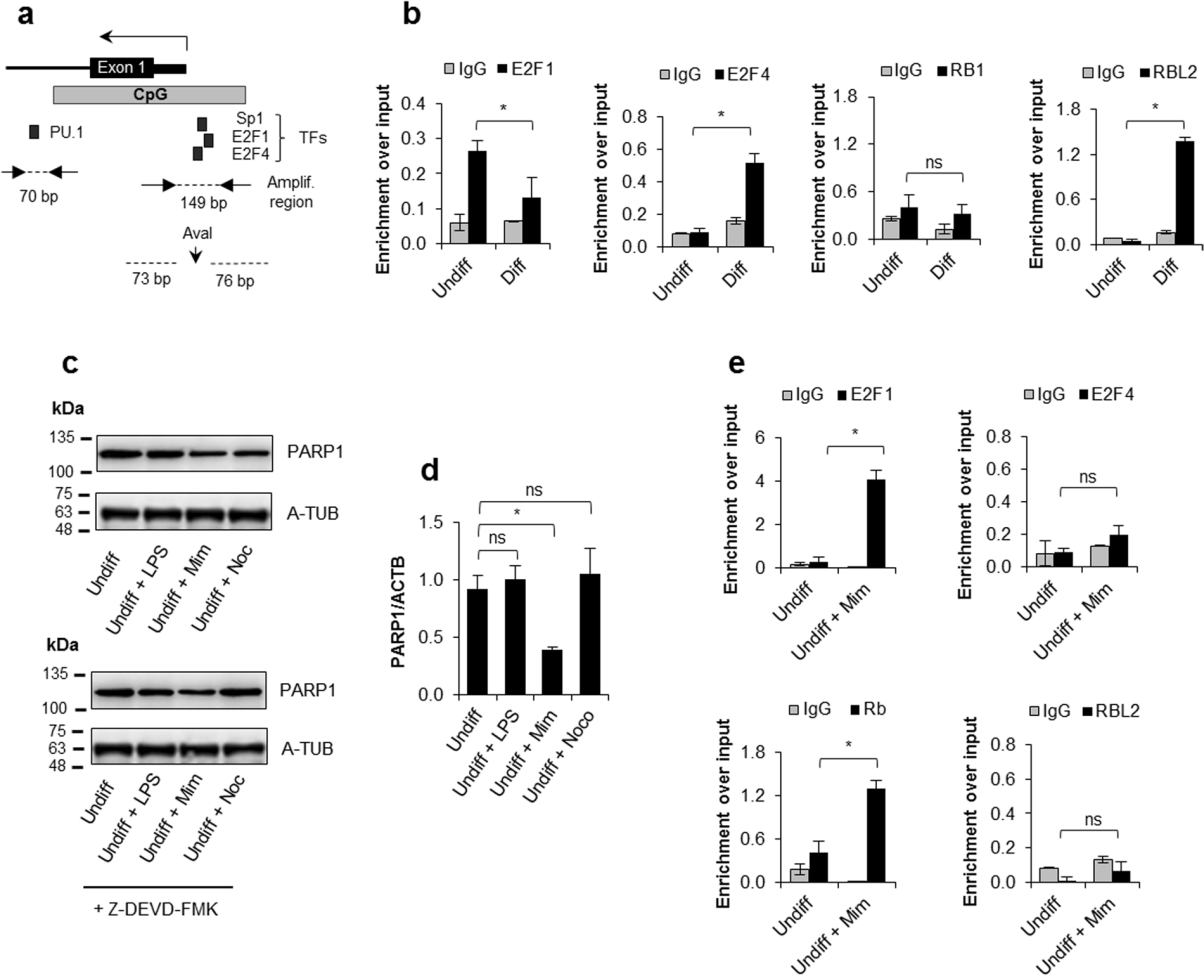
Repression of *PARP1* transcription is associated with cell cycle exit to G0 and the recruitment of RBL2 protein to *PARP1* promoter. Panel (a) shows the graphic representation of *PARP1* regulatory region adjacent to TSS, which spans POLR2A and H3K27ac according to the ENCODE. Small black boxes indicate the binding sites of transcription factors, which are considered as possibly involved in the regulation of *PARP1* transcription in hematopoietic stem and progenitor cells and monocytes. The association of E2F1 and E2F4 transcription factors as well as RB1 and RBL2 proteins with *PARP1* promoter in undifferentiated and differentiated THP-1 cells was determined with ChIP (**b**). The effect of LPS (100 ng/ml, 24 h) and cell cycle inhibitors (phase specific arrest in G1 and G2 was induced by cell treatment with 200 μM mimosine (Mim) and 0.5 μM nocodasol (Noco) for 48 h) on PARP1 protein level was monitored with western blot (upper **c**). In order to eliminate the impact of caspases, likely activated by cell cycle inhibitors, on the level of PARP1 protein LPS, mimosine and nocodasol were added in a mixture with caspase-3 and -7 inhibitor Z-DEVD-FMK (10 μM) (lower c). PARP1 mRNA was quantified in cells treated with LPS, Mim and Noco using *real-time* PCR (**d**). The recruitment of E2F1, E2F4, RB1 and RBL2 to *PARP1* promoter in cells arrested in G1 phase was determined with ChIP (**e**).

The progression of oesophageal adenocarcinoma carcinogenesis is associated with stepwise overexpression of miR-223 which targets PARP1 mRNA. Therefore, the differentiation process which changes the miRNA pattern might provide monocytes with PARP1-specific miRNA for RISC processing of the PARP1 template^13,18,19^. To verify this hypothesis, we utilized cell-permeable Argonaute-2 (AGO2, RISC catalytic component) inhibitor – aurintricarboxylic acid (iAgo2)^20^. To confirm that RISC activity is inhibited by selected iAgo2, PARP1-expressing THP-1 cells were transfected with PARP1 siRNA (which uses the RISC complex for the silencing) in both the presence and absence of iAgo2. As shown in Supplem. Figure 2a, iAgo2 decreased the silencing efficiency of delivered siRNA considerably and maintained the PARP1 mRNA level. However, treatment of monocytes with validated iAgo2 inhibitor did not affect either the PARP1 protein level or PARP1 mRNA in human monocytes (Supplem. Figure 1b,c). Also, in THP-1 cells, the contribution of RISC to the decrease in PARP1 mRNA in differentiated cells was not confirmed (Supplem. Figure 2d). Therefore, we excluded miRNA-mediated PARP1 mRNA degradation as a mechanism leading to a reduction in the PARP1 transcript in human monocytes and we subsequently focused on the chromatin-based mechanisms underlying the possible inhibition of *PARP1* transcription.

Since the extent of *PARP1* transcription is conditioned by the differentiation process, we first focused on the identification of possible *PARP1* gene enhancer(s)^21^. Knowing that *PARP1* is repressed in monocytes we searched for chromatin regions in the ± 600 kbp window upstream and downstream from the TSS, which are enriched in H3K27me3 and H3K4me1. We aligned ChIP-seq data for CD34+ Cultured Cells and CD14+ Primary Cells (GSM669907 and GSM1102793 for H3me1 and GSM537656 and GSM1102785 for H3K27me3) using Genome Data Viewer. Additionally, we included H3K4me3 (GSM537629 and GSM1102797), which marks active promoters, to distinguish between gene enhancers and promoters. First, we chose four regions from GeneHancer database of genome-wide enhancer-to-gene associations, embedded in GeneCards. However, predicted *PARP1* enhancers were not enriched in H3K4me1 in HPSCs nor a considerable increase in H3K27me3 in monocytes was observed (Supplem. Figure 2e). Whenever H3K27me3 was present in monocytes, it overlapped with gene promoters (Prom 2–4) or correlated with the loss of H3K4me3 (Prom 1).

**Figure 3:**
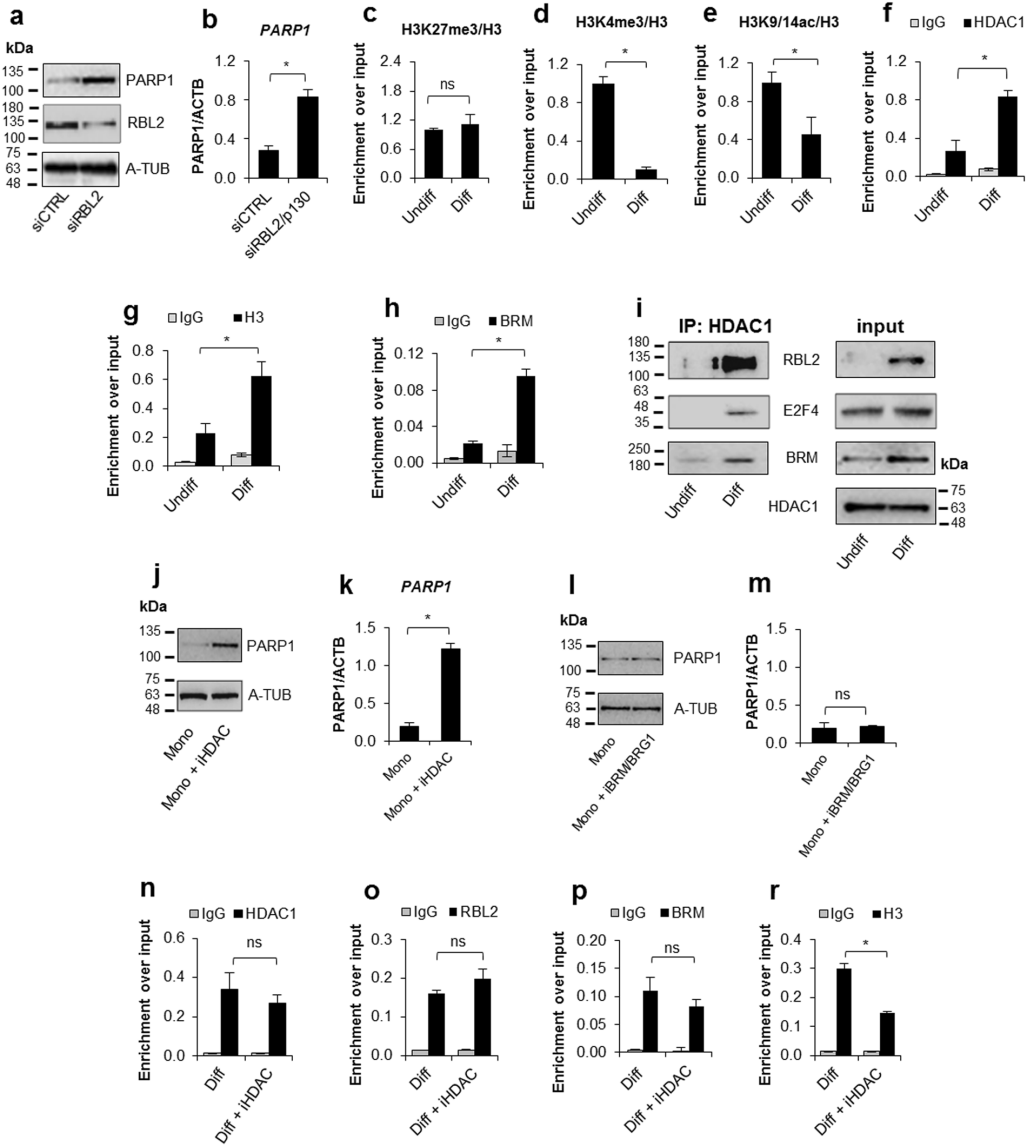
Quiescent state of differentiated cells entails the assembly of RBL2-E2F4-HDAC1-BRM repressor complex at *PARP1* promoter and the HDAC1-BRM induced chromatin compaction. The effect of RBL2 silencing on *PARP1* expression was determined with western blot (**a**) and *real-time* PCR (**b**) in human blood-derived monocytes 72 h after cell transient transfection with siRNA. Transcription regulating epigenetic marks (**c**,**d**,**e**) as well as HDAC1 (**f**), histone H3 density (**g**) and BRM (**h**) at *PARP1* promoter were analyzed with ChIP. The interaction among repressive complex components was confirmed with immunoprecypitation (**i**) by pulling down HDAC1 and detection of co-immunoprecipitated proteins with western blot. In order to verify the contribution of HDACs (**j**,**k**) and BRM (**l**,**m**) in *PARP1* repression blood-derived monocytes were incubated with the corresponding inhibitors (iHDAC – sodium butyrate – 0.5 mM and iBRM/iBRG1 – PFI-3 – 1 µM) for 24 h. *PARP1* expression was monitored at the protein level with western blot (**j**,**l**)), while PARP1 mRNA was determined with *real-time* PCR (**k**,**m**). The effect of iHDAC on HDAC1, RBL2 and BRM association with chromatin (**n**,**o**,**p**) and the chromatin compaction (H3 density) in differentiated cells was assayed with ChIP (**r**).

Therefore, using the UCSC Genome Browser (GRCh37/hg19), we created a simplified map of the *PARP1* promoter (Fig. 2a). We marked previously described regulatory elements as well as the monocyte relevant transcription factor PU.1 (overexpressed in monocytes; Fig. 1k,l, Supplem. Figure 3f,g) and E2F transcription factors, which show high binding probability (similarity between a registered sequence for the transcription factor binding sites and the input sequence > 0.89 according to TFBIND) and dependence on cell cycle progression.

To verify the specificity of further ChIP-qPCR experiments for the binding site of SP1 and E2F, which are located in the GC-rich region, we amplified this region in the corresponding PCR reaction, sequenced the PCR product (Supplem. Figure 3a) and digested it with AvaI (Supplem. Figure 3b). The lengths of digested and undigested products were in agreement with the predicted DNA fragments.

Since *PARP1* transcriptional repression was previously associated with DNA methylation of the *PARP1* promoter in response to toxic agents^1,11^, we first assessed if such a mechanism may also apply to differentiating monocytes, as DNA methylation and demethylation were documented to regulate cell type-specific gene transcription^22^. Despite treating of the proliferating THP-1 cells with the inhibitor of DNA methyltransferases, 5-azacytidine (iDNMT), before differentiation, the resulting differentiated cells did not express the greater extent of the PARP1 protein and mRNA than control, iDNMT untreated cells (Supplem. Figure 3c,d). It suggests that CpG island methylation does not contribute to a decrease in *PARP1* transcription in monocytes. As transcription of *IL1β* was documented to be regulated by promoter DNA methylation and, hence, was sensitive to iDNMT, we used this gene as a positive control (Supplem. Figure 3e)^23^.

Because PU.1 controls transcription in a cell type-specific manner and can act as a repressor of individual genes^24^, we performed the ChIP experiment to evaluate whether this transcription factor binds to the *PARP1* promoter in differentiated cells (Supplem. Figure 3h). Despite the high abundance of PU.1 in monocytes and differentiated THP-1 cells (Fig. 1k and l, Supplem. Figure 3f and g), this transcription factor was relatively weakly recruited to the *PARP1* promoter (black bars, left axis). On the contrary, the *IL1β* distal promoter (positive control) was strongly enriched in PU.1 in differentiated cells (grey bars, right axis). Further silencing of PU.1 in monocytes did not affect *PARP1* transcription (Supplem. Figure 3i and j), thus leading us to the conclusion that PU.1 is not responsible for the decrease in *PARP1* expression.

Other possible mechanisms were documented in the culture of rat and primary human cells, where *PARP1* transcription was repressed in the post-confluent cell culture by contact inhibition and followed by degradation of the SP1 transcription factor which controlled PARP1 expression^12^. Bearing in mind that the cell cycle exit is a prerequisite in certain types of differentiation, we tested if SP1 might be involved in *PARP1* regulation in HPSCs and monocytes. As shown in Supplem. Figure 3k and 3l, SP1 was equally expressed in both cell types. Furthermore, the differentiation process did not affect the association of SP1 with the *PARP1* promoter (Supplem. Figure 3m). We finally excluded the role of SP1 in transcriptional control of PARP1 in THP-1, by loading proliferating THP-1 cells with WP631, which intercalates GC-rich DNA fragments^25^. The WP631 induced a displacement of SP1 (Supplem. Figure 3n) from the *PARP1* promoter, but did not decrease *PARP1* transcription (Supplem. Figure 3o and p).

### Differentiation-associated loss of self-renewal potential entails E2F4 and RBL2 recruitment to the PARP1 promoter

**Figure 4:**
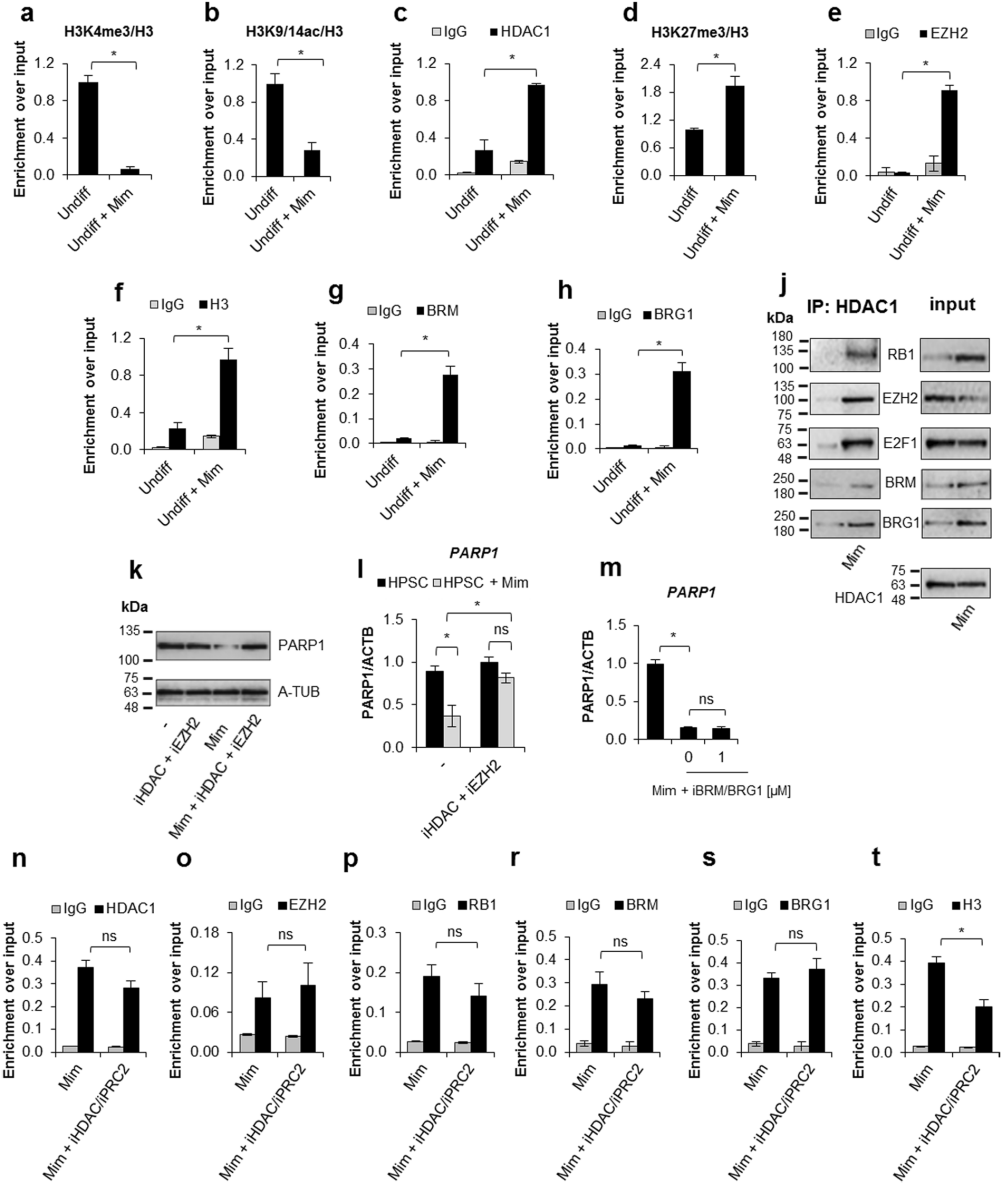
PRC2 facilitates *PARP1* silencing by associating with RB1-E2F1-HDAC1-BRM/BRG1 repressor complex in G1 arrested cells. Epigenetic modifications (**a**,**b** and **d**), recruitment of HDAC1 (**c**), EZH2 (**e**), BRM (**g**), BRG1 (**h**) to *PARP1* promoter and the density of histone H3 (**f**) were compared by ChIP between proliferating cells and cells arrested in G1 phase by their treatment with mimosine (Mim, 200 μM) for 48 h. Composition of *PARP1* repressive complex in G1-arrested cells was determined by pulling down HDAC1 and followed by WB detection of RB1, E2F1, EZH2, BRM and BRG1 (**j**). The expression of *PARP1* in HSPC supplemented with mimosine (50 µ) and additionally with 5 mM iHDAC, 125 nM iEZH2 and the mixture of 5 mM iHDAC and 125 nM and iEZH2 for 24 h was analyzed with western blot (**k**) and *real-time* PCR (**l**). Expression of *PARP1* in G1-arrested cells treated with BRM/BRG1 inhibitor (iBRM/iBRG1 - PFI-3 – 1 μM) was determined with *real-time* PCR (**n**). Changes in epigenetic marks and in protein composition in *PARP1* promoter upon THP-1 cells treatment with iHDAC/iEZH2/Mim versus Mim alone were monitored with ChIP (**n**–**t**).

Following the data which linked *PARP1* downregulation with cell growth arrest, we tested the association of E2F1 and E2F4 transcription factors with the corresponding binding sites in the *PARP1* promoter in proliferating and differentiated THP-1 cells (Fig. 2b). These transcription factors were reported to bind negative cell cycle regulators belonging to the retinoblastoma family: RB1/p105, RBL1/p107 and RBL2/p130^26^. The ChIP experiment revealed that E2F1, which was bound to chromatin in proliferating cells, was displaced with E2F4 during differentiation. However, the expression of these proteins remained unchanged (Fig. 3i – input, Fig. 4j – input). According to the published data, E2F4 interacts with RBL2 to repress human cell cycle-dependent genes in G0-arrested cells^27^. Although PARP1 has not been directly linked with cell proliferation, enrichment of the *PARP1* promoter in E2F4 was followed by the binding of RBL2, and further analysis of cell proliferation confirmed that human blood-derived monocytes exit the cell cycle and, in contrast to HPSCs, remain quiescent (Supplem. Figure 4a). Of note, RBL2 expression was also increased in differentiated cells (Fig. 3i – input).

To verify if PARP1 repression occurs regardless of the cell cycle phase of arrested cells, we used undifferentiated THP-1 cells whose differentiation phenocopied monocyte-like interdependence between the cell cycle exit and silencing of PARP1 transcription (Supplem. Figures 4b and 1b–d) because of their higher survival rate and stronger response to cell cycle inhibitors. For synchronisation in G1 and in early S phase cells were treated with mimosine, while for G2 they were treated with nocodazole. Additionally, we exposed the cells to a low concentration of LPS to evaluate if pathophysiological stimulation of monocyte precursors could influence *PARP1* transcription without affecting cell cycle progression. In contrast to G1- and G2-arrested cells, treatment with LPS did not cause a noticeable decrease in the PARP1 protein level (Fig. 2c). However, the simultaneous administration of caspase-3 and -7 inhibitor (Z-VAD-FMK) with nocodazole prevented PARP1 loss in G2-arrested cells (Fig. 2c, lower panel), thus suggesting that nocodazole-induced PARP1 cleavage by caspases. Hence only G1 arrest was accompanied by a decline in PARP1 protein level. Similarly, PARP1 mRNA was decreased only in mimosine-treated cells (Fig. 2d), which suggested that *PARP1* repressors in the myeloid cells might be limited to E2F-RB complexes, as PARP1 downregulation occurs before DNA replication. Moreover, the role of the RB protein family has been documented to control HSC proliferation and quiescence^28^, while E2F4 was shown to regulate HPSC commitment to lymphoid lineages^29^. However, in contrast to differentiated cells, the mimosine treatment resulted in the recruitment of even more E2F1 and RB1 to the *PARP1* promoter instead of E2F4-RBL2 (Fig. 2e). Our observation is in agreement with the paradigm that E2F1 preferentially interacts with RB1 and blocks S phase entry, while E2F4-RBL2 complexes are common in quiescent state and displace E2F1 during late differentiation^27,30^. The contribution of RBL2 to *PARP1* silencing in human monocytes and THP-1 differentiating cells was further confirmed by silencing of this protein, which substantially increased *PARP1* transcription (Fig. 3a and b, Supplem. Figure 4c and d).

### E2F4-RBL2-associated HDAC1 and BRM repress PARP1 transcription by condensing chromatin in human monocytes

Histone modifications determine the chromatin structure and DNA availability for transcription machinery. Thus, to negatively control gene transcription, E2F-RB complexes require additional components capable of nucleosome remodelling. The data collected up until now indicate that retinoblastoma proteins may interact with hBRM and BRG1 (SNF2/SWI2), which directly modify histone density, and with HDAC1 (histone deacetylase-1) and SUV39H1 (histone H3-K9 methyltransferase 1)^26^. The latter two compact the chromatin by histone deacetylation and trimethylation of histone H3 at lysine 9, respectively. To find other components of the E2F4-RBL2 repressor complex, we searched for differentiation-induced changes in the chromatin structure and, by using inhibitors to enzymes inserting or erasing particular histone modifications, we either confirmed or excluded their role in the regulation of *PARP1* transcription.

As is shown in Fig. 3c, the differentiation process was not followed by transcription-repressive histone H3 lysine 27 trimethylation (H3K27me3) but by a substantial reduction of transcription promoting H3 lysine 4 trimethylation (H3K4me3; Fig. 3d). Despite cell treatment with the inhibitors of KDM5B (Lysine Demethylase 5B) and LSD1 (Lysine-specific Histone Demethylase 1), which specifically remove methyl residues from tri- and di-/mono-methylated H3K4, respectively, *PARP1* remained repressed in both differentiated THP-1 and human monocytes (Supplem. Figure 4e–h). Therefore, demethylation of H3K4 was not confirmed to repress *PARP1* transcription during human monocyte differentiation. Likewise, the differentiation induced a decrease in another transcription-activating histone modification, i.e. acetylation (Fig. 3e), which in our study was simultaneously detected at lysine 9 and 14 of histone H3 (H3K9/14ac). Searching for the possible histone deacetylase, we found HDAC1, which was previously shown to interact with RB proteins, in the long list of *PARP1* promoter-interacting proteins of the Genome Browser database. Histone deacetylase was also observed to be stably bound to an E2F target promoter during early G1 in proliferating cells and released at the G1-S transition^31^. Immunoprecipitation of chromatin-bound HDAC1 revealed that the *PARP1* promoter is enriched in this enzyme in differentiated cells (Fig. 3f). Furthermore, cell differentiation was associated with the increase in histone H3 density (Fig. 3g) and the recruitment of BRM (SWI/SNF, Fig. 3h), but not BRG1 (Supplem. Figure 4i). Co-immunoprecipitation of HDAC1 provided further evidence that this deacetylase interacts with E2F4, RBL2 and BRM, forming the E2F4-RBL2-HDAC1-BRM repressor complex (Fig. 3i). Notably, neither expression of E2F4 nor E2F1 was considerably changed by differentiation (input Fig. 3i, input Supplem. Figure 4j).

Surprisingly, the inhibition of histone deacetylases in differentiating THP-1 cells with a pan-inhibitor, sodium butyrate (iHDAC), prevented *PARP1* repression (Supplem. Figure 4k and l) and completely restored PARP1 expression in human terminally differentiated monocytes (Fig. 3j and k). Although BRM and BRG1 are involved in histone remodelling and modulation of nucleosome density^32^, the inhibition of BRM/BRG1 in monocytes and differentiating THP-1 cells did not affect *PARP1* transcription (Figs 3l,m, and Supplem. Figure 4m).

The E2F4-RBL2-HDAC1 multiprotein functional entity had been documented to repress p73 in the osteosarcoma MDR HosDXR150 cell line and assembly at the E2F-regulated promoters of the quiescent synchronised 3T3 cells^33,34^, but now we could show that such a complex extended to BRM is involved in gene silencing during monocyte differentiation.

Bearing in mind that de-repression of genes silenced with E2F4-RBL2-HDAC1-BRM has not been reported yet, we utilised iHDAC to determine the chromatin changes that are required to reverse differentiation-induced repression of *PARP1* transcription. Although iHDAC was capable of re-establishing histone acetylation (Supplem. Figure 4m), the components of repressor complex HDAC1, RBL2 and BRM remained associated with chromatin, thus suggesting that they do not interfere directly with the transcription machinery and that they are recruited regardless of the histone acetylation status (Fig. 3n and o). The observation that RBL2 silencing restores *PARP1* transcription indicates that this protein most likely anchors, or at least stabilises, the entire repressive complex and makes RBL2-DNA interaction a requisite for HDAC1 and BRM recruitment. The lack of iBRM/iBRG1 effect on *PARP1* repression as well as the persistent association of BRM with the *PARP1* promoter after the re-establishment of histone acetylation, which led to decrease in histone density, may support the previous finding indicating that BRM recruitment to chromatin is in some cases a prerequisite to the binding of HDAC1^35^.

**Figure 5:**
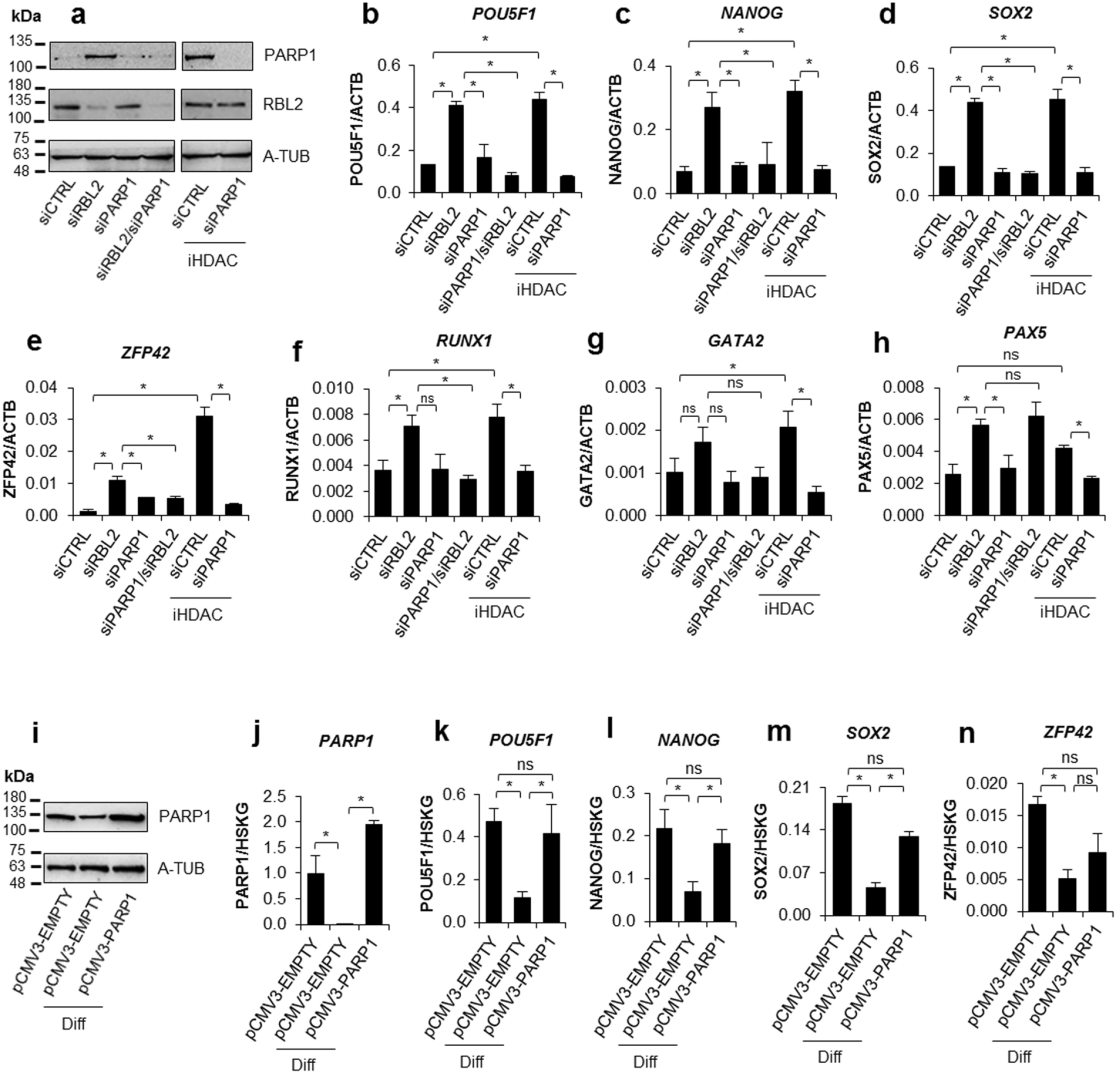
PARP1 protein recovery enhances transcription of pluripotency stem cell factors *POU5F1, NANOG and SOX2*. In order to recover *PARP1* transcription human monocytes were transfected with siRBL2, siPARP1 and siRBL2/siPARP1 or incubated with iHDAC for 48 h and additionally transfected with siPARP1. The PARP1 and RBL2 protein was determined with western blot (**a**). The expression of embryonic stem cell relevant transcription factors and *RUNX1, GATA2 and PAX5* was analyzed with *real-time* PCR (**b**–**h**). THP-1 cell were transfected with pCMV3-EMPTY vector (negative control) and pCMV3-PARP1 expression vector and differentiated with PMA (10 ng/ml) for 48 h. PARP1 level was monitored with western blot (**i**) and *real-time* PCR (**j**). *POU5F1*, *NANOG* and *ZFP42* transcription was quantified in genomic DNA-free samples using primers designed to span exon-exon boundary (Supplem. Table 1: POU5F1 ex-ex, NANOG ex-ex, ZFP42 ex-ex) by *real-time* PCR (**k**,**l**,**n**). The same method was employed to determine mRNA level of the intronless *SOX2* (**m**). For normalization of *POU5F1*, *NANOG, SOX2* and *ZFP42* transcription (**k**–**n**) three housekeeping genes (HSKG) were used (*ACTB*, *GAPDH*, *B2M*).

Several lines of evidence indicate that GC content is an essential hallmark of an open chromatin structure and that GC-rich promoters correlate with the level of nucleosome depletion, unless distinct repressive mechanisms occur, which may also apply to the CpG island overlapping *PARP1* promoter^35^. One such mechanism might involve histone deacetylation as, according to published data, transcription-promoting histone acetylation facilitated histone eviction at the transcription start site without a contribution of any particular enzymes and chromatin remodelling complexes^36^. In monocytes, the re-established histone acetylation caused considerable reduction in nucleosome density (Fig. 3r), which firmly and negatively correlated with PARP1 transcription (Supplem. Figure 5g).

With these data, we cannot exclude the participation of other proteins in the assembly of the functional repressive unit, but we underline the fact that RBL2, as well as HDAC1, are crucial for the repression of *PARP1* transcription in human monocytes and that the yield of *PARP1* transcription is solely controlled by histone acetylation.

### The E2F1-RB1-HDAC1- EZH(PRC2)-BRM/BRG1(SWI/SNF) multiprotein complex represses PARP1 transcription in G1-arrested cells

As cell treatment with mimosine induced arrest in the G1 phase and a decrease in *PARP1* transcription, we wondered whether the mechanism of observed gene repression remained the same as in differentiated cells.

Knowing that the *PARP1* promoter in G1-arrested THP-1 cells is occupied by the E2F1-RB1 heterodimer, and not by E2F4-RBL2, we first assessed if the chromatin architecture was influenced by such a replacement. Also here the promoter was characterised by the loss of transcription-activating histone modifications: H3K4me3 and H3K9/14ac (Fig. 4a and b).

Similarly, HDAC1 was strongly overrepresented around the E2F-binding motif in the arrested cells (Fig. 5c). However, the inhibition of deacetylase activity in mimosine-treated THP-1 cells was insufficient to restore or even upregulate *PARP1* transcription (Supplem. Figure 5a), thus suggesting that other or a more complex set of histone-remodelling enzymes represses *PARP1* expression in G1-arrested cells. We found H3K27me3 enrichment in response to mimosine administration (Fig. 4d). In the canonical view, this histone modification hallmarks silenced genes, although recent data link it with bivalent and active genes^37^. Up until today, only one methyltransferase, EZH2, which is a core component of polycomb repressor complex 2 (PRC2), has been reported to insert such modification into nucleosomes^38^. The ChIP experiment revealed the association of EZH2 with the *PARP1* promoter around the E2F binding site in G1-arrested cells (Fig. 4e), which was further followed by the increased histone density (Fig. 4f) and, surprisingly, by recruitment of BRM and BRG1 (Fig. 4g and h). Immunoprecipitation of HDAC1 confirmed the interaction of this enzyme with E2F1, RB1, EZH2, BRM and BRG1 (Fig. 4j). Nonetheless, the inhibition of solely EZH2 activity did not affect *PARP1* transcription (Supplem. Figure 5b and c). Knowing that the regulatory sequence of our gene is simultaneously occupied by HDAC1 and EZH2, we applied the mixture of corresponding inhibitors. Only the joint action of iHDAC1 and iEZH2 prevented *PARP1* repression induced by THP-1 cell cycle arrest in G1 (Supplem. Figure 5b and c), thus suggesting that trimethylation of histone H3 at lysine 27 supports histone deacetylation in *PARP1* silencing and that PRC2 assembles with E2F1-RB1-HDAC1 at the *PARP1* promoter. Since proliferating hematopoietic stem cells respond with growth inhibition to physiological (TGF-β, MIP-1α) and pathological (radiation, mutagenic agents, endogenous reactive metabolites) stimuli, we also arrested these cells in the G1 phase (Supplem. Figure 5d)^39,40^. Like THP-1 cells, monocyte precursors treated with mimosine were characterised by decreased *PARP1* expression and required the simultaneous administration of iHDAC and iEZH2 to restore *PARP1* transcription (Fig. 4k and l). Although BRM and BRG1 occupy the PARP1 promoter in G1 arrested cells, the administration of their inhibitor failed to increase *PARP1* transcription (Fig. 4m, Supplem. Figure 4m) suggesting that also in mimosine-treated cells post-translational histone modifications (H3K9/14 acetylation and H3K27 trimethylation) determine transcription of the studied gene.

As in differentiated cells, the reinstatement of active chromatin by treatment of G1-arrested cells with iHDAC and iEZH2 (Supplem. Figure 5f and g) did not affect the association of RB1, HDAC1, EZH2, BRM and BRG1 with the *PARP1* promoter (Fig. 4n–s), but reduced the density of the nucleosomes considerably (Fig. 5t). The question as to how PRC2 components are attracted to the *PARP1* promoter still remains open, as according to our data histone deacetylation is not a prerequisite to sustaining PRC2, BRM and BRG1 associated with the chromatin. According to published data, some PRC2 subunits (JARID2 and PCL) target the complex to the unmethylated CpG island^41,42^. However, PRC2 does not contribute to *PARP1* silencing in differentiated cells, thus suggesting that another factor, most likely one of the E2F1-RB dimers or SWI/SNF complex components, anchors PRC2 at the *PARP1* promoter in G1-arrested cells. From our observations, what is most important is that histone deacetylation and trimethylation of H3K27 are not mutually redundant in repressing *PARP1* transcription and that PRC2 may serve as an active component of the E2F1-RB1-HDAC1-BRM-BRG1-based repressive complex. The joint action of HDAC1 and Polycomb repressive complexes has been documented at the promoter of p16^INK4a^ in senescent cells, but in that case, the H2.0-like homeobox 1 (HLX1) recruited chromatin modifiers^43^.

The question on why BRG1 binds *PARP1* promoter together with BRM1 remains open since these two enzymes were documented to play antagonistic roles in the differentiation^35^. The study on osteocalcin promoter revealed that BRG1 complexes activated the gene, while concurrently present BRM repressed transcription, was a prerequisite to HDAC1 binding and overrode activity of BRG1. It may suggest that the presence of a BRG1 primes *PARP1* promoter and serves as a mechanism, which ensures re-activation of gene transcription after removal of proliferation inhibiting factor.

### Downregulation of PARP1 transcription facilitates repression of pluripotent stem cell transcription factors in human monocytes

To verify whether PARP1 may sustain transcription of pluripotency stem cell genes and transcription factors, which are substantially suppressed in differentiated cells, we restored *PARP1* transcription in human monocytes via silencing of RBL2 as well as via their treatment with iHDAC. To exclude the PARP1-independent effect of RBL2 silencing and iHDAC administration, PARP1 was silenced with siRNA in the parallel samples (Fig. 5a, Supplem. Figure 6a and b). In the group of the seven studied transcription factors, expression of four of them, i.e. *POU5F1, NANOG, SOX2, ZFP42*, which are characteristic for embryonic stem cells, was increased in a PARP1-specific manner (Fig. 5b–e). The same pattern of interdependence between transcription of *PARP1* and pluripotency genes was observed in differentiating THP-1 cells (Supplem. Figure 6c–h), in which the up and downregulation of PARP1 protein was strictly followed by an alteration in transcription of *POU5F1, NANOG and SOX2*. Importantly, the minor effect of PARP1 recovery on the activation of *RUNX1*, *GATA2* or *PAX5* (Fig. 5f–h) was observed in monocytes since the increase in expression of these transcription factors in the presence of PARP1 remained relatively low or even insignificant (also *ZFP42* in THP-1 cells, Supplem. Figure 6i). Moreover, transcription of *PAX5* was found to be rather RBL2-dependent in monocytes since RBL2 single silencing or in combination with PARP1 silencing marginally, but significantly statistically upregulated the expression of this gene (Fig. 5h).

To maintain PARP1 protein level in differentiated cells and to limit the possible PARP1-independent, side effects of RBL2 silencing and HDACs inhibition on the transcription of *POU5F1, NANOG, SOX2 and ZFP42*, we transiently transfected proliferating THP-1 cells with pCMV3-PARP1 expression vector and differentiated them in parallel with cells transfected with pCMV3-EMPTY vector (Fig. 5i–n, Supplem. Figure 7a and b)^4^. Additionally, for POU5F1 and NANOG we used another set of primer pairs, which span exon-exon junction (Supplem. Figure 7c and d; cover all the transcript variants; in the case of POU5F1 also POU5F1P3) and the DNA was digested prior to reverse transcription.

As shown in Fig. 5i–j pCMV3-PARP1 vector abrogated differentiation-induced a decline of PARP1 abundance and sustained transcription of *POU5F1, NANOG and SOX2*, but not *ZFP42* (Fig. 5k–n). These data fully agree with the findings observed in differentiated cells, in which PARP1 level was maintained by the RBL2 silencing and by the inhibition of HDACs.

Simple PARP1 protein recovery in monocytes is likely insufficient to unlock expression of all PARP1-dependent genes, as the regulation of gene transcription is a rather multifactorial process and may require gene-specific chromatin activation before or simultaneously to PARP1 binding to gene regulatory sequence(s). However, even with only *POU5F1, NANOG* and *SOX2* we can support the assumption that *PARP1* repression in human monocytes may be a prerequisite for the suppression of particular pluripotency and that the PARP1 protein may act as a positive regulator of their transcription.

Quite the opposite situation was observed for promoters of genes encoding lineage-specific genes such as *IL1β* in osteoclasts, where PARP1 negatively regulated the transcription of this autocrine-acting cytokine and, therefore, inhibited osteoclast development^6^. It suggests that by acting in a promoter-specific manner, PARP1 may control the differentiation process in particular cell types dually, i.e. by sustaining expression of stem cells-specific transcription factors and repressing the differentiation-promoting factors. However, the specificity of PARP1 to gene regulatory elements remains unknown and may be equally linked to co-operation with particular transcription factors or chromatin status and histone tags. The fact that PARP1 has been documented to co-regulate gene transcription at many levels (e.g. PARylation of chromatin remodelling enzymes and CTCF insulator, histone exclusion) makes the whole picture even more complex^44^.

As PARP1 is involved in various intracellular processes, the possibility to modulate its expression might be of use in pharmacological interventions such as synthetic lethality^45^. As shown in Figs 3a,b,j,k, 4k,l knowledge on the molecular mechanism that determines *PARP1* transcription allows upregulation of *PARP1* expression by using the inhibitors of histone remodelers in both growth-arrested myeloid precursors as well as in differentiated cells. Therefore, our finding provides clues for a further search for *PARP1* silencing or overexpression in other cell types. Moreover, we show that *PARP1*-targeted interventions might be considered at different levels, i.e. promoter-associated chromatin writers and erasers as well as signaling cascades, because both HDAC and EZH2 inhibition as well as RBL2 silencing restored *PARP1* transcription.

Concluding, *PARP1* transcription is downregulated by RB-E2F-HDAC1-SWI/SNF-based complexes in a growth inhibition-dependent manner. By comparing expression of *PARP1* and *POU5F1*, *NANOG*, *SOX2*, *ZFP42* between differentiated monocytes and their proliferating precursors, as well as by restoring PARP1 expression in monocytes, we provide the functional link between expression of *PARP1* and pluripotency transcription factors in monocytes.

## Materials and Methods

RosetteSep™ Monocytes Enrichment Cocktail was purchased from STEM Cell Technologies (Tominex, Suchy Dwór, Poland), cell culture media from Biowest (CytoGen, Zgierz, Poland), recombinant human Stem Cell Factor (SCF), interleukin 3 (IL3) and interleukin 6 (IL6) from Peprotech (London, UK), phorbol 12-myristate 13-acetate (PMA), WB antibodies: anti-PARP1 (sc-7150), anti-PU.1 (sc-22805), anti-β-tubulin (sc-5546), siPARP1 (sc-29437), siPU.1 (sc-36330) were from Santa Cruz Biotechnology (AMX, Lodz, Poland), TRI Reagent, iAgo2 (aurintricarboxylic acid), mimosine, iHDAC (sodium butyrate), iDNMT (5-azacytidine), anti-E2F4 antibody (05–312), iBRM/BRG1 (PFI-3), anti-rabbit IgG (A0545) or anti-mouse IgG (A4416) (whole molecule)–peroxidase antibody produced in goat were from Sigma Aldrich (Poznan, Poland), ChIP grade antibodies: anti-histone H3 (#4620), anti-H3K4me3 (#9751), anti-H3K27me3 (#9733), anti-PARP1 (#9532), anti-SP1 (#9389), anti-E2F1 (#3742), anti-Rb (#9313), anti-RBL2 (#13610), anti-PU.1 (#2258), normal rabbit IgG (#2729) were purchased from Cell Signaling Technology (LabJOT, Warsaw, Poland), anti-acetyl-histone H3 (Lys9 + Lys14) (PA5-16194), anti-HDAC1 (PA1-860), siRBL2 (#AM16708), human CD14 antibody PE-Cy7® conjugate (A15852), Lipofectamine RNAiMAX, StemPro CD34+ Cells kit (A14059), Dynabeads™ Protein G, High-Capacity cDNA Reverse Transcription Kit, Click-iT® Nascent RNA Capture Kit for gene expression analysis, SuperSignal™ West Pico Chemiluminescent Substrate, OptiMem, AvaI, BigDye® Terminator v3.1 Cycle Sequencing Kit and Hi-Di formamide, DNA-free™ DNA Removal Kit were from Thermofisher Scientific (Thermofisher Sceintific, Warsaw, Poland). iLSD1 (SP2509), iEZH2 (UNC199), iKDMB5 (PBIT), WP 631 were from Cayman Europe, while Advanced TC™ culture plates from Geiner Bio-One (Biokom, Janki/Warsaw, Poland). Kapa Sybr Fast qPCR Master Mix and KAPA HiFi™ HotStart ReadyMix (2X) were purchased from Kapa Biosystems (Polgen, Łódź, Poland). EvaGreen® Dye, 20X in water was purchased from Biotium (Corporate Place Hayward, USA). NucleoSpin® Gel and PCR Clean-up Columns were from Macherey-Nagel (Düren, Germany), while ExTerminator from A&A Biotechnology (Gdynia, Poland). NucleoSpin® DNA RapidLyse was purchased from AQUA LAB (Warsaw, Poland), Human PARP1 Gene cDNA Clone (full-length ORF Clone), espression ready, untagged (HG11040-UT; pCMV3-PARP1) and pCMV3-untagged Negative Control Vector (CV011; pCMV3-EMPTY) from Hölzel Diagnostika Handels GmbH (Köln, Germany), ViaFect™ Transfection Reagent, QuantiFluor® RNA and dsDNA System from Promega (Warsaw, Poland).

### Monocyte isolation and cell culture

Human monocytes were isolated using RosetteSep™ Monocytes Enrichment Cocktail from the buffy coat derived from healthy donors in Blood Bank in Lodz. The purity of isolated cells was confirmed by flow cytometry (LSR II BD Flow Cytometer) after cell staining with PE-Cy7® conjugated anti-CD14 antibody. Monocytes were cultured in RPMI supplemented with 10% FBS and penicylin/streptomycin solution (50 U/ml and 50 µg/ml, respectively) at 37 °C, 5% CO_2_. The study as well as the processing of buffy coat and human-derived monocytes was approved by the Bioethical Committee at University of Lodz no 19/KBBN-UŁ/I/2016 and all methods were performed in accordance with the relevant guidelines and regulations. Human Hematopoietic Progenitor/Stem cells (HPCs, CD34+) were cultured in StemPro®−34 SFM basal liquid medium supplemented with StemPro®−34 Nutrient Supplement and the mixture of recombinant human SCF, IL3, IL6 (100 ng/ml, 50 ng/ml, 50 ng/ml, repectively) to maintain pluripotency and cell proliferation.

To track the *PARP1* repression during differentiation THP-1 cells (monocytic leukemia/premonocytes) differentiated with PMA (10 ng/ml) for 48 h were used as a model. Cells were cultured in RPMI supplemented with 10% FBS and penicylin/streptomycin solution (50 U/ml and 50 µg/ml, respectively) at 37 °C, 5% CO_2_.

### Western blot

Depending on the primary antibody, membranes were incubated with anti-rabbit or anti-mouse peroxidase = conjugated antibody (Sigma Aldrich). Signal was developed using SuperSignal™ West Pico Chemiluminescent Substrate and acquired with ChemiDoc-IT2 (UVP, Meranco, Poznan, Poland).

### Gene expression

RNA was extracted with TRI Reagent. In parallel to RNA, the double stranded DNA was quantified using Quantus™ Fluorometer and corresponding QuantiFluor® RNA and dsDNA Systems to exclude possible contamination of RNA with genomic DNA. The concentration of double stranded DNA was always below the detection limit. The isolated RNA was reversed transcribed (High Capacity cDNA Reverse Transcription Kit, Thermofisher Scientific) and cDNA was quantified by *real-time* PCR (Mic, Bio Molecular Systems) using primers listed in Supplem. Table 1 and Kapa Sybr Fast qPCR Master Mix. *ACTB* was used as a reference gene.

### Quantification of nascent RNA

The synthesis of *PARP1* mRNA was measured using Click-iT® Nascent RNA Capture Kit according to manufacturer instructions. Undifferentiated and differentiated cells were fed with ethylene uridine (0.2 mM) for 12 h. 1 µg of the pulled down RNAs was reverse transcribed with High Capacity cDNA Reverse Transcription Kit and the copy number of *PARP1* and *ACTB* was quantified with *real-time* PCR (Mic, Bio Molecular Systems) using Kapa Sybr Fast qPCR Master Mix and corresponding primers listed in Supplementary Table 1.

### Gene silencing

For transient gene silencing cells were transfected with siRNA. Briefly, siRNA:Lipofectamine RNAiMAX complexes (0.6 nmol siRNA per 1 µl of transfection reagent) were prepared in serum free OptiMEM medium and added to cells cultured in full RPMI growth medium. The efficiency of the mRNA and protein knock-down was monitored by *real-time* PCR and Western blot, respectively.

For the gene silencing in monocytes freshly isolated cells were allowed to attach (Advanced TC™ culture plates, Geiner Bio-One) for 3 h prior to culture supplementation with siRNA:Lipofectamine RNAiMAX. THP-1 were transfected 24 h before initiation of the differentiation with PMA.

To determine the efficiency of aurintricarboxylic acid (iAgo2) inhibition of RISC cells were incubated with this compound (10 µM) for 2 h prior to transfection with siPARP1. Cells were harvested 48 h after transfection for the quantification (*real-time* PCR) of PAPR1 expression.

### Cell treatment with cell-cycle inhibitors

In order to induce the cell cycle arrest in G1/early S and G2/M phase proliferating THP-1 cells were incubated with 200 µM mimosine and 0.5 µM nocodasole, respectively for 48 h. Hematopoietic progenitor cells were arrested in G1/early S phase by the treatment with 50 µM mimosine for 48 h.

### The cell-cycle analysis

The distribution of cells in different phases of the cell cycle was analyzed with flow cytometry. Briefly, proliferating and differentiated cells as well as cells treated with the cell cycle inhibitors and LPS (100 ng/ml) were fixed with 1% formaldehyde in PBS, permeabilized with 0.1% Triton-X100 in PBS, digested with RNAse (5 ug/ml) in PBS and stained with propidium iodide (5 ug/ml). The cell fluorescence was measured with flow cytometry (LSR II BD Flow Cytometer) and acquired data was processed with FlowJo X software.

### Cell treatment with inhibitors of DNA and histone-modifying enzymes

For the evaluation of the contribution of DNA methylation in *PARP1* repression proliferating THP-1 cells were first fed with 1 µM DNMT inhibitor – 5-azacytidine for 48 h and then differentiated with 10 ng/ml PMA for the following 48 h.

Freshly isolated monocytes were allowed to attach (Advanced TC™ culture plates, Geiner Bio-One) for 3 h. After that, cells were incubated with HDAC inhibitor (iHDAC, sodium butyrate, 0.5 mM) for the following 24 h. The differentiating THP-1 cells were supplemented with iHDAC (5 mM) 24 h after addition of PMA and cells were further incubated with the HDAC inhibitor for 24 h (total differentiation time remained unchanged).

THP-1 cells arrested in G1/early S phase were added with the mixture of iHDAC/iEZH2 (5 mM and 125 µM, respectively) 24 h after arrest induction and cultured for the following 24 h. For the culture of hematopoietic progenitor cells arrested with minosine iHDAC was used at the concentration of 0.5 mM, while iEZH2 at 100 µM.

### Co-immunoprecipitation

Cells were lyzed in IP buffer (20 mM HEPES pH = 7.9, 75 mM KCl, 2.5 mM MgCl_2_, 0.1% NP-40) and 1 mg of total cell lysate was incubated with 5 µg of anti-HDAC1 rabbit antibody for at 4 °C 3 h. 10 µl of Dynabeads*™* Protein G was added for the last 1 h of the immunoprecipitation. Beads were washed five times with the IP buffer, suspended in 50 µl of RIPA buffer and added with 6x SDS loading buffer. After heating at 75 °C for 10 min beads were removed on magnetic stand and immunoprecipitated proteins were separated on 8% SDS-gel.

### Chromatin immunoprecipitation

The immunoprecipitation of selected proteins likely associated with *PARP1* promoter was carried out according to Saccani *Nat. Immunol*. 3, 69–75 (2002). In brief, cells were cross-linked with 1% formaldehyde solution, isolated chromatin was sheared with the ultrasonic homogenizer Bandelin Sonopuls (HD 2070). After overnight incubation with antibody-conjugated magnetic beads (Dynabeads*™* Protein G) the immunoprecipitated chromatin was washed and decrosslinked overnight at 65 °C. The DNA was isolated with phenol:chlorophorm:isoamyl alcohol and analyzed with *real-time* PCR. The immunoprecipitated fragment of DNA, which spans PU.1 binding site was quantified with Kapa Sybr Fast qPCR Master Mix, while GC rich fragment spanning SP1/E2F binding sites was quantified with KAPA HiFi™ HotStart ReadyMix supplemented with EvaGreen® Dye and 7% DMSO. The list of primers used for *PARP1* promoter are listed in Suppl. Table 1.

### AvaI digestion

The specificity of amplified GC rich DNA fragment was confirmed by the digestion of this region with AvaI according to the instruction provided by manufacturer. The digested and undigested templates were separated on the polyacrylamide gel in TBE buffer, stained with propidium iodide solution (5 μM) and the pictures were acquired with ChemiDoc-IT2 (UVP, Meranco, Poznan, Poland).

### DNA sequencing

After cleaning on spin columns (NucleoSpin® Gel and PCR Clean-up) PCR products were extended using the BigDye® Terminator v3.1 Cycle Sequencing Kit (4 μl of BigDye® mix, 30 ng of primer and 50–70 ng of the amplicon). Extended products were cleaned on spin columns (ExTerminator), dried in a Speed-Vac system, re-suspended in 20 μl of Hi-Di formamide and analyzed using ABI Prism 3130™ Genetic Analyzer. Sequences were edited and analyzed using FinchTV software.

### Transient cell transfection

Proliferating THP-1 cells were transfected with pCMV3-EMPTY and pCMV3-PARP1 expression vector using ViaFect™ Transfection Reagent. In brief, 0.1 µg DNA was mixed with 0.8 µl ViaFect™ Transfection Reagent in OptiMem, incubated for 15 min at room temperature and added to 200 000 proliferating THP-1 cells. After 24 h cells were differentiated with PMA (10 ng/ml) for another 48 h. To confirm transfection, DNA was isolated from whole cells using NucleoSpin®DNA RapidLyse Kit according to manufacturer instruction. After protein digestion and Proteinase K heat inactivation, cell lysate was treated with RNAse (4 µg) for 10 min at room temperature. To confirm cell transfection with vectors isolated DNA was sequenced using pCMV3 and PARP1 primers (Supplem. Table 1, Supplem. Figure 7).

RNA was extracted with TRI Reagent, but was additionally treated with DNA-free™ DNA Removal Kit prior to reverse transcription.

### Statistical analysis

Bars in the figures represent mean ± standard error of the mean (SEM). Student’s t-test was used to compare the difference between two means (*P < 0.05). All bars represent data from three independent biological replicates (with two technical replicates each).

## Electronic supplementary material

Supplementary information

## References

[1] Hottiger MO (2015). Nuclear ADP-Ribosylation and Its Role in Chromatin Plasticity, Cell Differentiation, and Epigenetics. Annu. Rev. Biochem..

[2] Rosado MM, Bennici E, Novelli F, Pioli C (2013). Beyond DNA repair, the immunological role of PARP-1 and its siblings. Immunology.

[3] Heinz S (2010). Simple combinations of lineage-determining transcription factors prime cis-regulatory elements required for macrophage and B cell identities. Mol. Cell.

[4] Olah G (2015). Differentiation-Associated Downregulation of Poly(ADP-Ribose) Polymerase-1 Expression in Myoblasts Serves to Increase Their Resistance to Oxidative Stress. PLoS One.

[5] Bauer M (2011). Human monocytes are severely impaired in base and DNA double-strand break repair that renders them vulnerable to oxidative stress. Proc. Natl. Acad. Sci. USA.

[6] Robaszkiewicz A (2016). ARTD1 regulates osteoclastogenesis and bone homeostasis by dampening NF-kappaB-dependent transcription of IL-1beta. Sci. Rep..

[7] Gao F, Kwon SW, Zhao Y, Jin Y (2009). PARP1 poly(ADP-ribosyl)ates Sox2 to control Sox2 protein levels and FGF4 expression during embryonic stem cell differentiation. J. Biol. Chem..

[8] Hemberger M (2003). Parp1-deficiency induces differentiation of ES cells into trophoblast derivatives. Dev. Biol..

[9] Roper SJ (2014). ADP-ribosyltransferases Parp1 and Parp7 safeguard pluripotency of ES cells. Nucleic Acids Res..

[10] Gong C (2012). Methylation of PARP-1 promoter involved in the regulation of nano-SiO2-induced decrease of PARP-1 mRNA expression. Toxicol. Lett..

[11] Gao A (2010). Methylation of PARP-1 promoter involved in the regulation of benzene-induced decrease of PARP-1 mRNA expression. Toxicol. Lett..

[12] Zaniolo K (2005). Regulation of the poly(ADP-ribose) polymerase-1 gene expression by the transcription factors Sp1 and Sp3 is under the influence of cell density in primary cultured cells. Biochem. J..

[13] Streppel MM (2013). MicroRNA 223 is upregulated in the multistep progression of Barrett’s esophagus and modulates sensitivity to chemotherapy by targeting PARP1. Clin. Cancer Res..

[14] Qin Z (2012). The use of THP-1 cells as a model for mimicking the function and regulation of monocytes and macrophages in the vasculature. Atherosclerosis.

[15] Mintz PJ (2014). Exploiting human CD34+ stem cell-conditioned medium for tissue repair. Mol. Ther..

[16] Boland MJ, Nazor KL, Loring JF (2014). Epigenetic regulation of pluripotency and differentiation. Circ. Res..

[17] Morey L, Santanach A, Di Croce L (2015). Pluripotency and Epigenetic Factors in Mouse Embryonic Stem Cell Fate Regulation. Mol. Cell Biol..

[18] Ivey KN, Srivastava D (2010). MicroRNAs as regulators of differentiation and cell fate decisions. Cell Stem Cell.

[19] Martinez NJ, Gregory RI (2010). MicroRNA gene regulatory pathways in the establishment and maintenance of ESC identity. Cell Stem Cell.

[20] Tan GS (2012). Small molecule inhibition of RISC loading. ACS Chem. Biol..

[21] Ong CT, Corces VG (2011). Enhancer function: new insights into the regulation of tissue-specific gene expression. Nat. Rev. Genet..

[22] Moore LD, Le T, Fan G (2013). DNA methylation and its basic function. Neuropsychopharmacology.

[23] Wessels I, Fleischer D, Rink L, Uciechowski P (2010). Changes in chromatin structure and methylation of the human interleukin-1β gene during monopoiesis. Immunology.

[24] Wei F, Zaprazna K, Wang J, Atchison ML (2009). PU.1 can recruit BCL6 to DNA to repress gene expression in germinal center B cells. Mol. Cell Biol..

[25] Mansilla S, Priebe W, Portugal J (2004). Sp1-targeted inhibition of gene transcription by WP631 in transfected lymphocytes. Biochemistry.

[26] Giacinti C, Giordano A (2006). RB and cell cycle progression. Oncogene.

[27] Litovchick L (2007). Evolutionarily conserved multisubunit RBL2/p130 and E2F4 protein complex represses human cell cycle-dependent genes in quiescence. Mol. Cell.

[28] Viatour P (2008). Hematopoietic stem cell quiescence is maintained by compound contributions of the retinoblastoma gene family. Cell Stem Cell.

[29] Enos ME, Bancos SA, Bushnell T, Crispe IN (2008). E2F4 modulates differentiation and gene expression in hematopoietic progenitor cells during commitment to the lymphoid lineage. J. Immunol..

[30] Paramio JM, Segrelles C, Casanova ML, Jorcano JL (2000). Opposite functions for E2F1 and E2F4 in human epidermal keratinocyte differentiation. J. Biol. Chem..

[31] Ferreira R (2001). Cell cycle-dependent recruitment of HDAC-1 correlates with deacetylation of histone H4 on an Rb-E2F target promoter. EMBO Rep..

[32] Tang L, Nogales E, Ciferri C (2010). Structure and function of SWI/SNF chromatin remodeling complexes and mechanistic implications for transcription. Prog. Biophys. Mol. Biol..

[33] La Sala D (2003). Triggering of p73-dependent apoptosis in osteosarcoma is under the control of E2Fs-pRb2/p130 complexes. Oncogene.

[34] Rayman JB (2002). E2F mediates cell cycle-dependent transcriptional repression *in vivo* by recruitment of an HDAC1/mSin3B corepressor complex. Genes Dev..

[35] Flowers S, Nagl NG, Beck GR, Moran E (2009). Antagonistic roles for BRM and BRG1 SWI/SNF complexes in differentiation. J. Biol. Chem..

[36] Di Cerbo V (2014). Acetylation of histone H3 at lysine 64 regulates nucleosome dynamics and facilitates transcription. Elife.

[37] Young MD (2011). ChIP-seq analysis reveals distinct H3K27me3 profiles that correlate with transcriptional activity. Nucleic Acids Res..

[38] Kuzmichev A (2002). Histone methyltransferase activity associated with a human multiprotein complex containing the Enhancer of Zeste protein. Genes Dev..

[39] Parmar K, D’Andrea AD (2012). Stressed out: endogenous aldehydes damage hematopoietic stem cells. Cell Stem Cell.

[40] Wright EG, Pragnell IB (1992). Stem cell proliferation inhibitors. Baillieres Clin. Haematol..

[41] Hunkapiller J (2012). Polycomb-like 3 promotes polycomb repressive complex 2 binding to CpG islands and embryonic stem cell self-renewal. PLoS Genet..

[42] Pasini D (2010). JARID2 regulates binding of the Polycomb repressive complex 2 to target genes in ES cells. Nature.

[43] Martin N (2013). Interplay between Homeobox proteins and Polycomb repressive complexes in p16INK(4)a regulation. EMBO J..

[44] Schiewer MJ, Knudsen KE (2014). Transcriptional roles of PARP1 in cancer. Mol. Cancer Res..

[45] Aly A, Ganesan S (2011). BRCA1, PARP, and 53BP1: conditional synthetic lethality and synthetic viability. J. Mol. Cell Biol..

