# Author Correction: Downregulation of *PARP1* transcription by promoter-associated E2F4-RBL2-HDAC1-BRM complex contributes to repression of pluripotency stem cell factors in human monocytes

**DOI:** 10.1038/s41598-018-23162-3

**Published:** 2018-04-05

**Authors:** Ewelina Wiśnik, Tomasz Płoszaj, Agnieszka Robaszkiewicz

**Affiliations:** 1 Department of Biophysics of Environmental Pollution, Institute of Biophysics, University of Lodz, Pomorska 141/143, 90-236 Lodz, Poland; 2 Department of Molecular Biology, Medical University of Lodz, Narutowicza 60, 90-136 Lodz, Poland; 3 Department of General Biophysics, Institute of Biophysics, University of Lodz, Pomorska 141/143, 90-236 Lodz, Poland

Correction to: *Scientific Reports* 10.1038/s41598-017-10307-z, published online 25 August 2017

The original version of this Article contained errors in the spelling of the authors Ewelina Wiśnik, Tomasz Płoszaj & Agnieszka Robaszkiewicz which were incorrectly given as Wiśnik Ewelina, Płoszaj Tomasz & Robaszkiewicz Agnieszk.

These errors have now been corrected in the HTML and PDF versions of the Article, and in the accompanying Supplementary Information document.

