# Supplementary material for: Downregulation of PARP1 transcription by promoter-associated E2F4-RBL2-HDAC1-BRM complex contributes to repression of pluripotency stem cell factors in human monocytes
Source: Sci Rep. 2017 Aug 25;7:9483. doi: 10.1038/s41598-017-10307-z (PMC5572705; doi:10.1038/s41598-017-10307-z)
Supplement: Supplementary file 1 — Supplementary information [file 41598_2017_10307_MOESM1_ESM.pdf]

Downregulation of *PARP1* transcription by promoter-associated E2F4-RBL2-HDAC1-BRM complex contributes to repression of pluripotency stem cell factors in human monocytes

Ewelina Wiśnik[1], Tomasz Płoszaj[2], Agnieszka Robaszkiewicz[3]*

[1] Department of Biophysics of Environmental Pollution, Institute of Biophysics, University of Lodz, Pomorska 141/143, 90-236 Lodz, Poland

[2] Department of Molecular Biology, Medical University of Lodz, Narutowicza 60, 90-136 Lodz, Poland

[3] Department of General Biophysics, Institute of Biophysics, University of Lodz, Pomorska 141/143, 90-236 Lodz, Poland

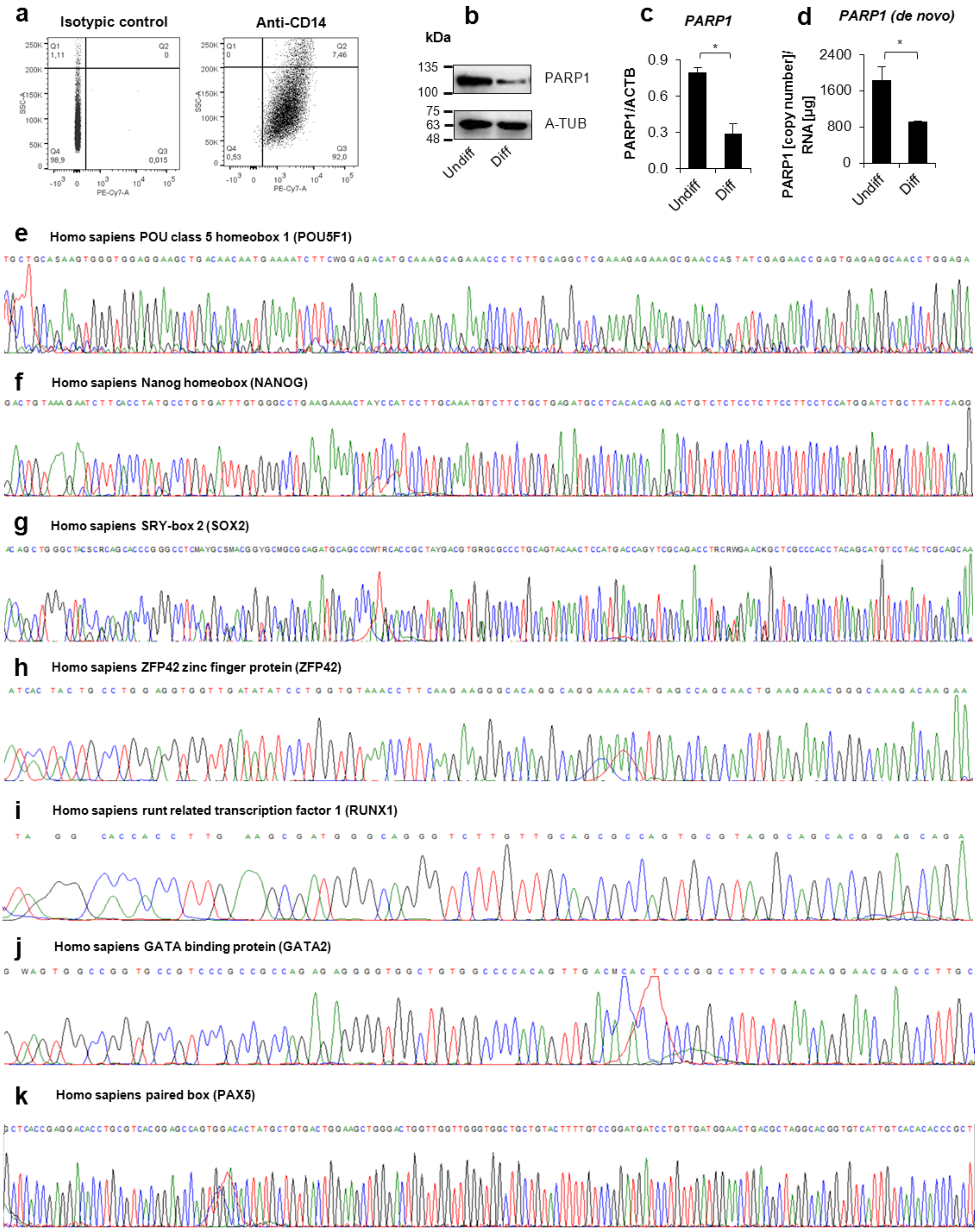

Supplementary Figure 1

Purity of blood-derived human monocytes was verified by their staining anty-CD14-PE-Cy7 antibody followed by fluorescence analysis with flow cytometry. Measurement of PARP1 protein level (b) and mRNA (c) in THP-1 cells differentiated with PMA (10 ng/ml, 48 h) was conducted using western blot and *real-time* PCR, respectively. Accumulation of nascent PARP1 mRNA was measured using Click-iT® Nascent RNA Capture Kit (Thermofisher Scientific) (d). (e-k) To validate quantitative *real-time* PCR data for transcription factors detected in HPSCs the amplified cDNA was stained with sequencing kit and analysed using ABI Prism 3130™ Genetic Analyser. Sequences were edited and analyzed using FinchTV software.

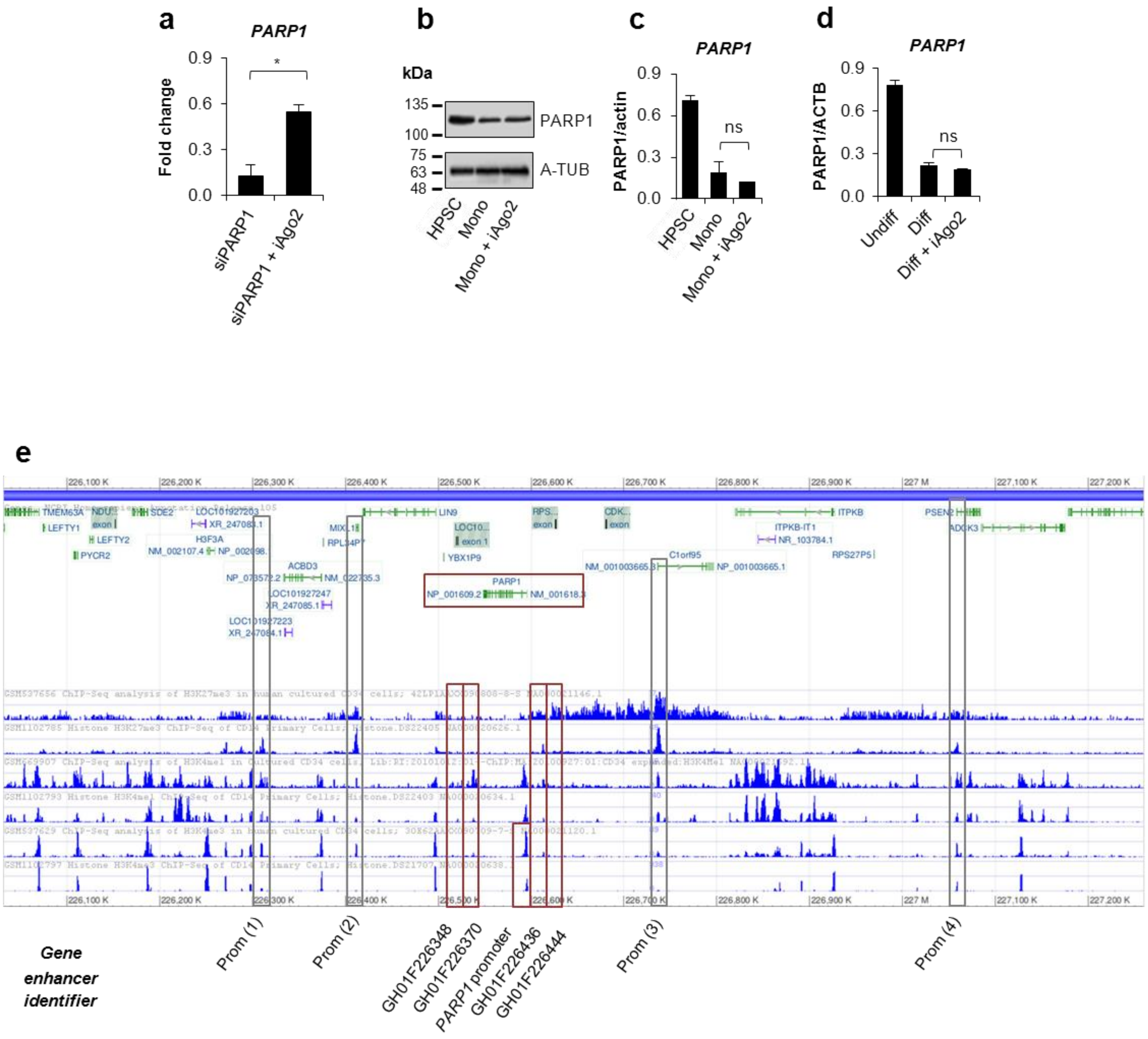

Supplementary Figure 2

The efficiency of aurintricarboxylic acid (iAgo2, 10 μM) to inhibit RISC activity was determined based on PARP1 mRNA degradation in cells transfected with siPARP1 (a). The quantity of PARP1 transcript was normalised to the corresponding level in non-transfected cells. The contribution of RISC to *PARP1* repression in monocytes was monitored by comparing PARP1 protein (b) and mRNA (c) in monocytes and THP-1 (d) cells treated and untreated with the inhibitor of AGO2 (iAgo2 - 10 μM, 48 h). (e) To search for potential *PARP1* enhancers in the window ± 0.7 Mbp from *PARP1* TSS the H3K4me1, H3K27me3 and H3K4me3 histone marks, which correspond to enhancer, inactive enhancers and active promoters, respectively, were compared using ChIP-seq data for CD34+ Cultured Cells and CD14+ Primary Cells (GSM669907 and GSM1102793 for H3me1, GSM537656 and GSM1102785 for H3K27me3, GSM537629 and GSM1102797 for H3K4me3) from Human Epigenome Atlas 9 and were aligned in Genome Data Viewer. Red boxes mark four regions from GeneHancer database of genome-wide enhancer-to-gene associations, embedded in GeneCards. Grey boxes mark gene promoters high in H3K27me3 in monocytes and H3K4me3 in HSPCs.

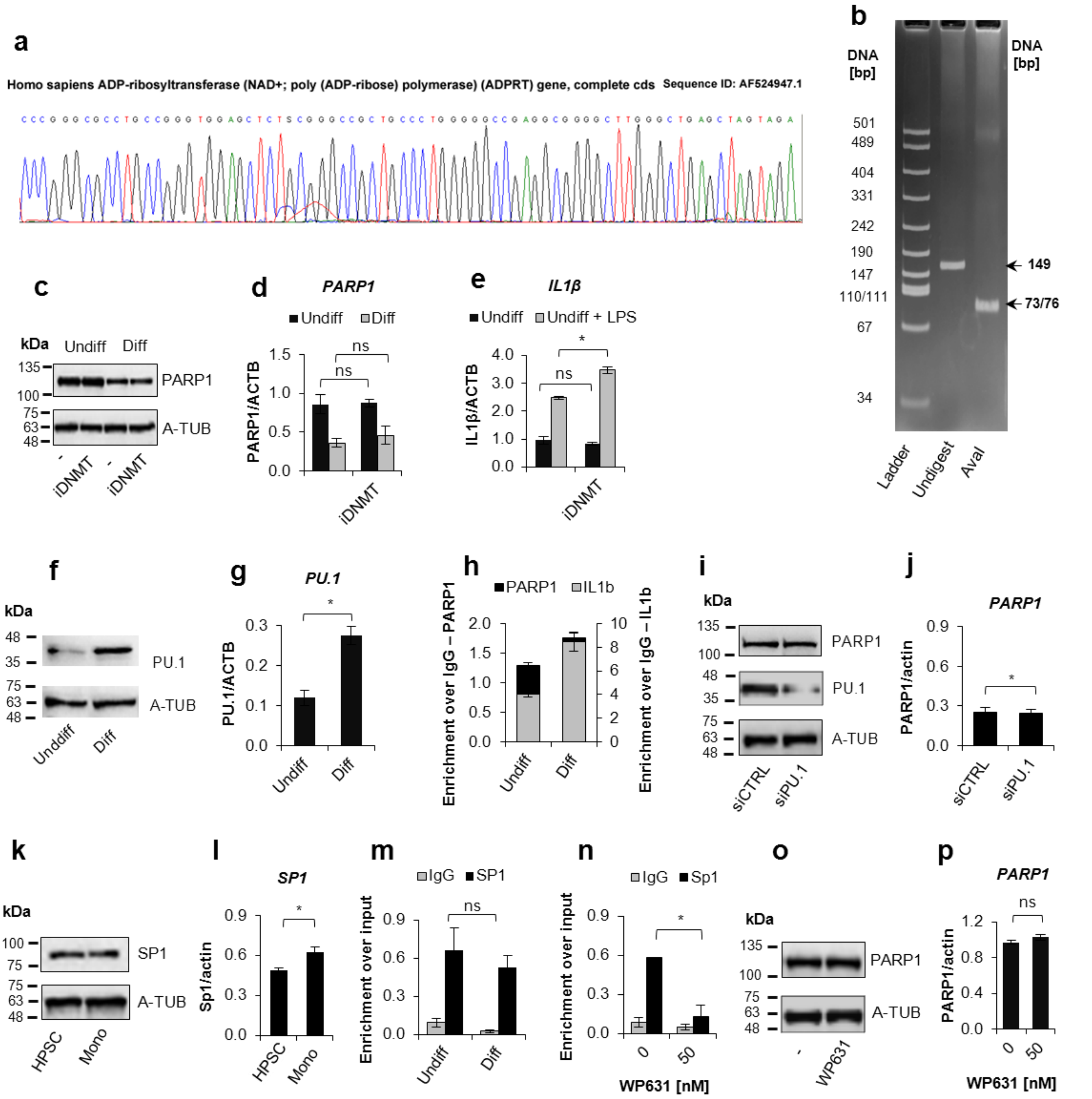

Supplementary Figure 3

Validity of *real-time* PCR reaction, which follows immunoprecipitation of *PARP1* promoter-associated proteins, was further confirmed by the sequencing of pulled-down and amplified DNA fragment (a). The fidelity/specificity of the *real-time* PCR reaction carried out on the immunoprecipitated fragments of DNA high in GC was verified by digestion of amplified fragment with AvaI followed by polyacrylamide gel electrophoresis (b). The contribution of DNA methyltransferases in *PARP1* repression was verified by comparing the level of PARP1 protein (c) and mRNA (d) between THP-1 cells loaded and not with iDNMT prior to differentiation (10 µM, 48 h). iDNMT sensitive *IL1β* was used as a positive control (e). PU.1 expression in undifferentiated and differentiated THP-1 cells was monitored with western blot (f) and *real-time* PCR (g). Association of PU.1/SPI-1 with *PARP1* promoter was analysed with ChIP-qPCR (h, black bars, left axis). As a positive control, we used distal promoter of human *IL1β*, which is characterised by strong recruitment of PU.1 in differentiated THP-1 cells (h, grey bars, right axis). The effect of PU.1 silencing on *PARP1* expression in human monocytes is shown with the western blot in panel (i) and *real-time* PCR in panel (j). The level of SP1 protein (k) and mRNA (l) was compared between HSPC and blood-derived monocytes. The association of SP1 with the *PARP1* promoter in undifferentiated and differentiated THP-1 cells was monitored with ChIP (m). To displace SP1 from *PARP1* promoter THP-1 cells were treated with WP631 (50 nM) for 48 h and dissociation of SP1 was confirmed with ChIP (n). Cells treated and untreated with WP631 were analysed for PARP1 expression with western blot (o) and *real-time* PCR (p).

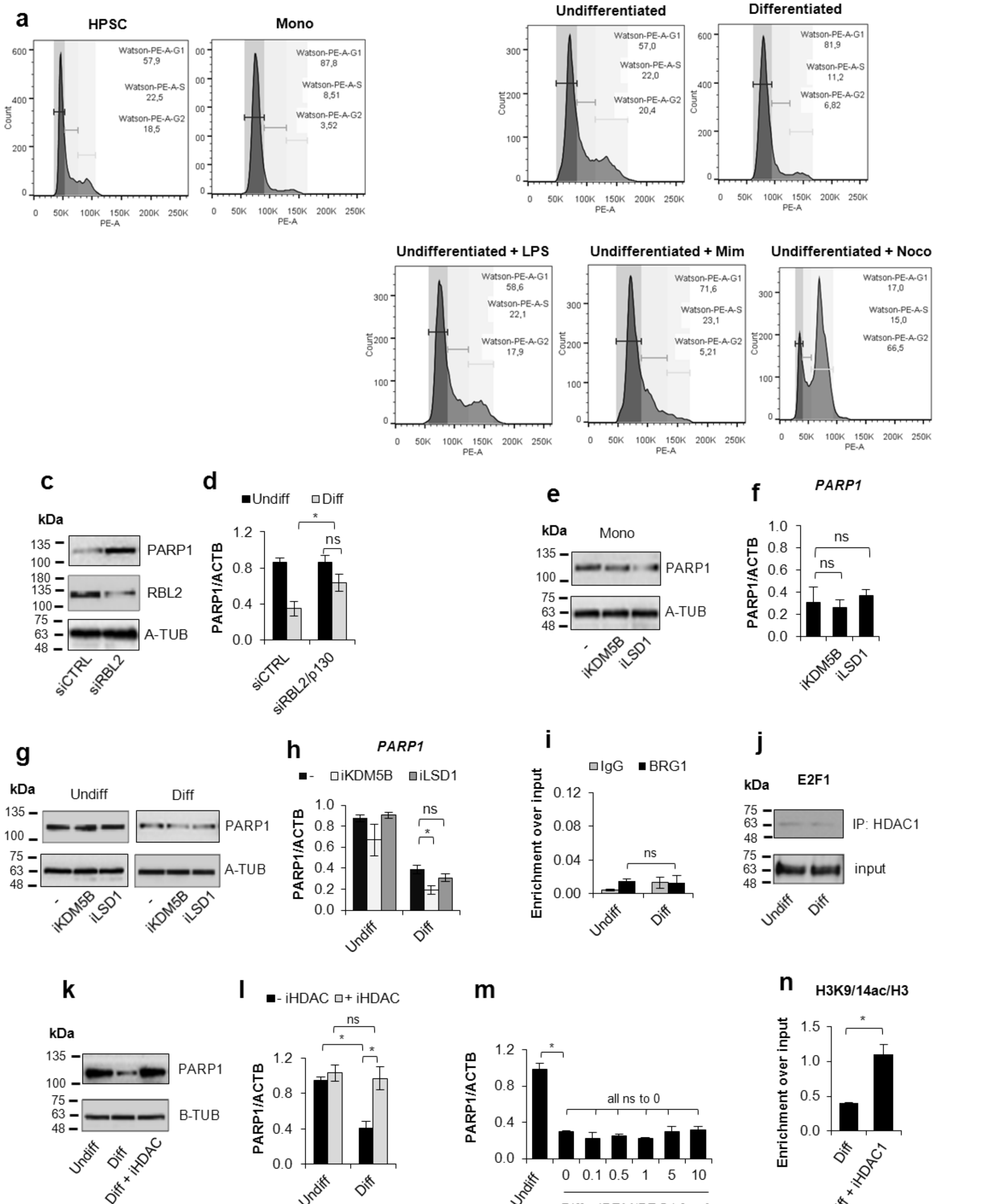

Supplementary Figure 4

Distribution of proliferating HSPCs and blood-derived monocytes in the cell cycle phases was analysed with flow cytometry using propidium iodide for the DNA staining (a). The same method was employed to monitor the cell cycle progression in undifferentiated and differentiated THP-1 (b). Additionally, THP-1 cells were treated with LPS (100 ng/ml) for 24 h. To induce phase specific arrest in G1 and G2 cells were treated with 200 µM mimosine (Mim) and 0.5 µM nocodasol (Noco), respectively for 48 h. Effect of RBL2 silencing on *PARP1* expression was determined with western blot (c) and *real-time* PCR (d) in THP-1 cells differentiated for 48 h. Transient cell transfection with siRNA was carried out 8 h prior to cell culture medium supplementation with PMA (10 ng/ml). To verify the contribution of H3K4me3 and H3K4me1/2 demethylases – KDM5B and LSD1, respectively, in *PARP1* silencing blood-derived monocytes (e, f) and differentiating THP-1 cells (g, h) were incubated with the corresponding inhibitors (iKDM5B – PBIT – 10 µM, iLSD1 – SP2509 – 5 µM) for 24 h. *PARP1* expression was monitored at the protein level with western blot (e, g), while PARP1 mRNA was determined with *real-time* PCR (f, h). BRG1 association with PARP1 promoter was determined by ChIP (i), while the interaction of E2F1 with repressor complex components by HDAC1 co-immunoprecipitation followed by E2F1 detection with western blot (j). Effect of HDAC inhibition on PARP1 protein (k) and mRNA (l) level was monitored in differentiating THP-1 cells (iHDAC – 5 mM was added to the culture 24 h before the end of differentiation). The effect of iBRM/iBRG1 inhibitor (PFI3) was studied by supplementing differentiating cells (24 h after PMA administration) with different concentrations of inhibitor (0.1 – 100 µM) and measuring mRNA level of PARP1 mRNA with *real-time* PCR (m). Re-establishment of histone acetylation at the *PARP1* promoter in differentiated cells by their supplementation with iHDAC (5 mM, 24 h) was confirmed with CHIP (n).

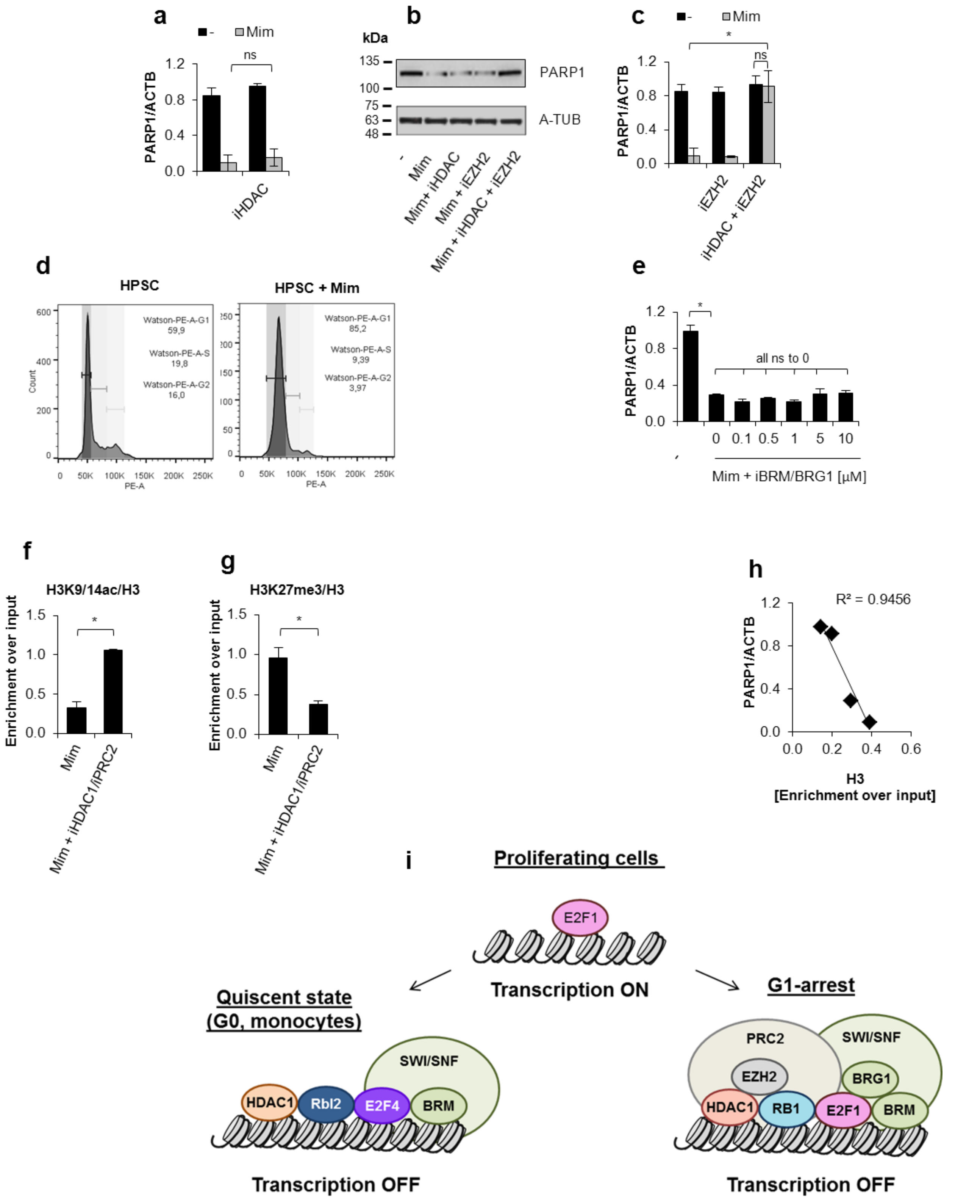

Supplementary Figure 5

Effect of iHDAC (5 mM) and iPRC2 (125 nM), as well as their combination on PARP1 protein (b) and mRNA (a, c) level, was monitored in THP-1 cells treated with mimosine (200 µM) for 48 h. Inhibitors were added to cell culture 24 h prior to the collecting samples. For analysis of the cell cycle in proliferating and mimosine (50 µ) treated HSPCs, cells were stained with propidium iodide (5 µM), and their fluorescence was read by flow cytometry (d). In (e) THP-1 cell were treated with iBRM/BRG1 (0.1 – 100 µM) 24 h after arresting cell growth with mimosine in order to verify the contribution of BRM/BRG1 in *PARP1* repression. Re-establishment of histone H3K9/14 acetylation and decrease in H3K27me3 at the *PARP1* promoter in mimosine (200 µM, 48 h) treated THP-1 cells, which were additionally supplemented with iHDAC/iPRC2 (5 mM/125 nM, 24 h) was confirmed by CHIP (f, g). The correlation between PARP1 mRNA (*real-time* PCR) and histone H3 density (ChIP) was computed using linear regression based on data acquired for untreated, differentiated and mimosine-treated cells (g). Graphical presentation of repressor complex components assembling at the *PARP1* promoter in differentiated and G1-arrested cells is shown in (i).

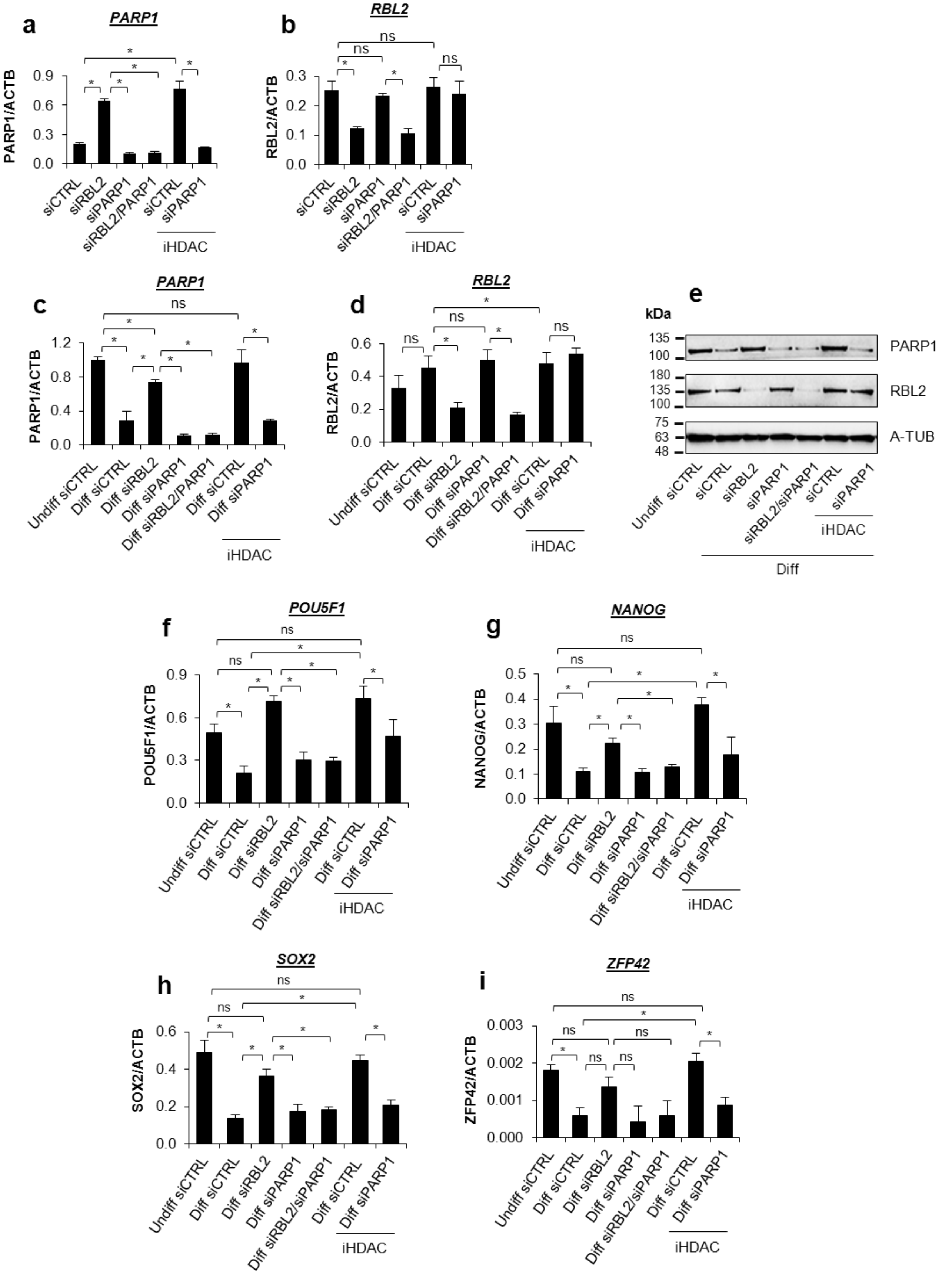

Supplementary Figure 6

The level of PARP1 (a, c) and RBL2 (b, d) mRNA in blood-derived monocytes (a, b) and THP-1 cells (c, d) transfected with siRNA was measured by *real-time* PCR. PARP1, RBL2 and α-tubulin (A-TUB) protein level in THP-1 cells was monitored by western blot (e). Expression (mRNA) of pluripotent stem cell transcription factors (f-i) in THP1 cells transfected with siRNA and treated with iHDAC to restore *PARP1* transcription was determined by *real-time* PCR.

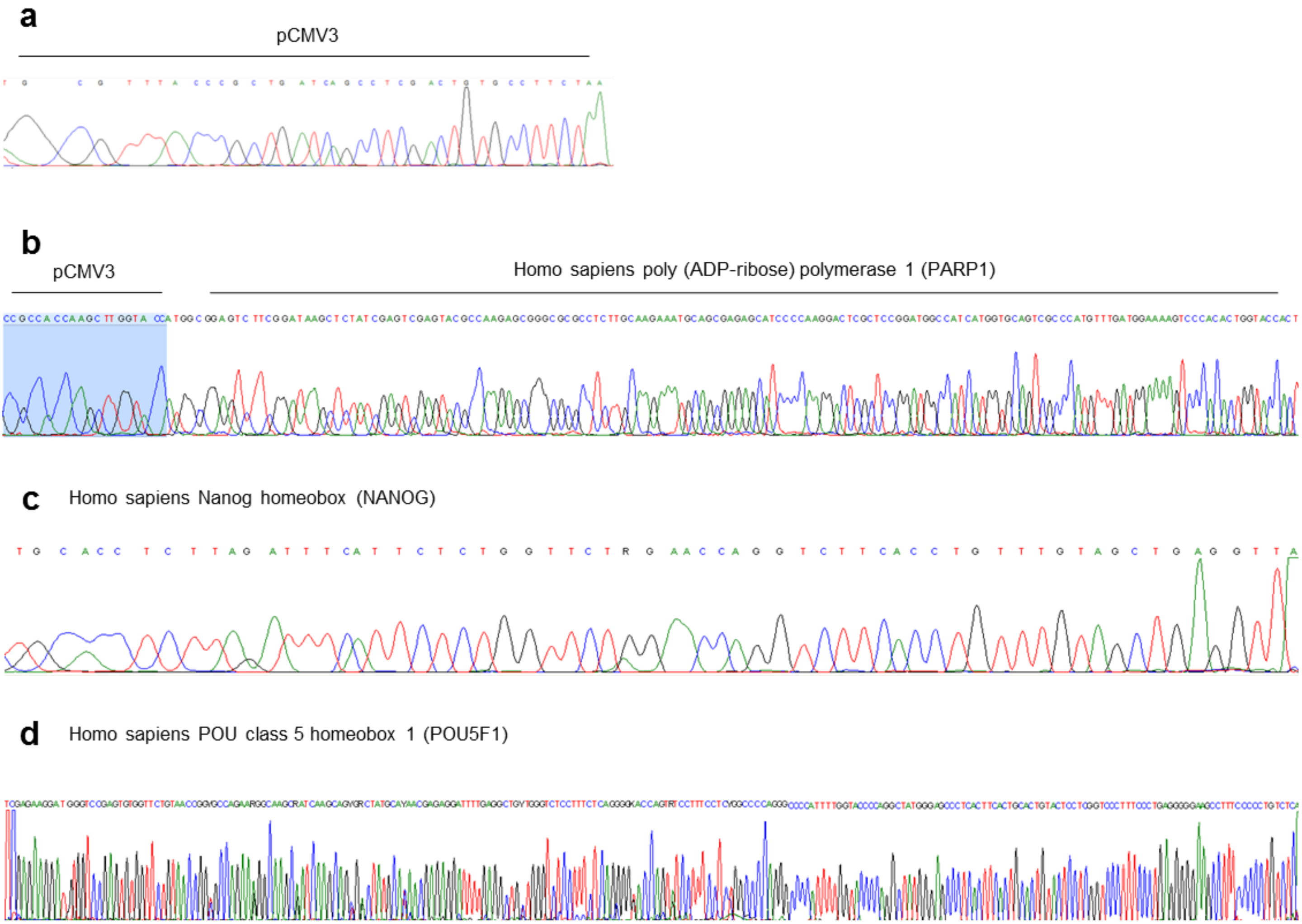

Supplementary Figure 7

DNA isolated from tranfected THP1 cells was sequenced to confirm the presence of control (pCMV3-EPMTY) and PARP1 coding vector (pCMV3-PARP1) using primers listed in Supplem. Table 1. Validity of POU5F1ex-ex and NANOGex-ex primers was confirmed by sequencing of amplified cDNA template (c and d). Any genomic and vector DNA was digested prior to reverse transcription.

Primers	Sequence (5'-3')
**Used for real-time PCR**	
PARP1 Fwd	AAGCCCTAAAGGCTCAGAACG
PARP1 Rev	ACCATGCCATCAGCTACTCGGT
ACTB Fwd	TGGCACCCAGCACAATGAA
ACTB Rev	CTAAGTCATAGTCCGCCTAGAAGCA
GAPDH Fwd	GGAGTCAACGGATTTGGTCGTA
GAPDH Rev	GGCAACAATATCCACTTTACCA
B2M Fwd	GACTTGTCTTTCAGCAAGGA
B2M Rev	ACAAAGTCACATGGTTCACA
RBL2 Fwd	AGAGGATGCTGAGGAGGAAA
RBL2 Rev	CAATAGCCTGGGTTGGATCT
SP1 Fwd	CCATACCCCTTAACCCCG
SP1 Rev	GAATTTTCACTAATGTTTCCCACC
PU.1 Fwd	CAGCTCTACCGCCACATGGA
PU.1 Rev	TAGGAGACCTGGTGGCCAAGA
NANOG Fwd	CATGAGTGTGGATCCAGCTTG
NANOG Rev	CCTGAATAAGCAGATCCATGG
POU5F1 Fwd	CTCACCCTGGGGGTTCTATT
POU5F1 Rev	CTCCAGGTTGCCTCTCACTC
SOX2 Fwd	GCACATGAACGGCTGGAGCAACG
SOX2 Rev	TGCTGCGAGTAGGACATGCTGTAGG
ZFP42 ex-ex Fwd	TCACAGTCCAGCAGGTGTTTG
ZFP42 ex-ex Rev	TCTTGTCTTTGCCCGTTTCT
PAX5 Fwd	GAGCGGGTGTGTGACAATGA
PAX5 Rev	GCACCGGAGACTCCTGAATAC
RUNX1 Fwd	CTGCTCCGTGCTGCCTAC
RUNX1 Rev	AGCCATCACAGTGACCAGAGT
GATA2 Fwd	GCAAGGCTCGTTCCTGTTC
GATA2 Rev	GGTTCTGCCCATTCATCTTG
pCMV3 Fwd	TAATACGACTCACTATAGGG
pCMV3 Rev	TAGAAGGCACAGTCGAGG
POU5F1 ex-ex Fwd	CGAGAAGGATGTGGTCCGAG
POU5F1 ex-ex Rev	GAGACAGGGGGAAAGGCTTC
NANOG ex-ex Fwd	AACCTCAGCTACAAACAGGTGA
NANOGex-ex Rev	TCTGCGTCACACCATTGCTA
**Used for ChIP**	
PU.1 binding site Fwd	AAACACCGCCACCCAGAAAGG
PU.1 binding site Rev	AGCTGGTGTTGCCTTTCGCT
E2F/RB binding site Fwd	AACGCCCACGGAACCC
E2F/RB binding site Rev	CTACTAGCTCAGCCCAAGCC

Supplementary Table 1

List of primers used for gene expression and ChIP-qPCR
